# Design and application of volatilizable solid additives in non-fullerene organic solar cells

**DOI:** 10.1038/s41467-018-07017-z

**Published:** 2018-11-07

**Authors:** Runnan Yu, Huifeng Yao, Ling Hong, Yunpeng Qin, Jie Zhu, Yong Cui, Sunsun Li, Jianhui Hou

**Affiliations:** 1 Beijing National Laboratory for Molecular Sciences, State Key Laboratory of Polymer Physics and Chemistry, CAS Research/Education Center for Excellence in Molecular Sciences, Institute of Chemistry, Chinese Academy of Sciences, Beijing, 100190 China; 2 University of Chinese Academy of Sciences, Beijing, 100049 P. R. China

## Abstract

Most of the high-performance organic solar cells are fabricated with the assistance of high-boiling-point solvent additives to optimize their charge transport properties; this has adverse effects on the OSCs’ stability and reproducibility in large-scale production. Here, we design volatilizable solid additives by considering the molecular structure feature of an acceptor–donor–acceptor-type non-fullerene acceptor. The application of solid additives can enhance the intermolecular π–π stacking of the non-fullerene acceptor and thus facilitate the charge transport properties in the active layers, leading to improved efficiencies of OSCs. Importantly, devices fabricated using volatilizable solid additives exhibit higher stability and reproducibility when compared with the OSCs processed with solvent additives. Our results not only demonstrate an approach of applying volatilizable solid additives to benefit the large-scale production of OSCs but also provide a potential direction for designing specific solid additives for different active layers.

## Abstract (web summary)

High-boiling-point solvent additives are commonly used to optimize the device performance of organic solar cells but they make problems for device stability and reproducibility. Here Yu et al. design volatilizable solid additives that can improve the device performance without causing above issues.

## Introduction

Organic solar cells (OSCs) have desirable advantages in that they are low cost, flexible and lightweight^1–4^, and their power conversion efficiencies (PCEs) have rapidly increased in recent years^5–9^. As charge generation, transport and recombination in bulk-heterojunction (BHJ) OSCs are highly dependent on the nanoscale morphology of active layers, a few processing methods such as use of solvent additives and thermal/vapor annealing were developed to control the blend morphology. Solvent additives such as 1,8-diiodooctane (DIO)^10,11^, diphenyl ether^12^ and 1-phenylnaphthalene^13^ have dominant advantages for improving photovoltaic performance but have been shown to be unfavorable for the devices’ stability and reproducibility^14–18^.

Recently, non-fullerene (NF) acceptors, especially small molecular acceptors comprising acceptor–donor–acceptor (A–D–A) structures, have gradually replaced fullerene acceptors, with PCEs above 13% having been achieved^19–25^. Unlike fullerene derivatives, the A–D–A acceptors have planar conjugated backbones and bulky alkyl chains substituted on their central units. Since the side chains have strong steric hindrance to impede ordered intermolecular stacking, the π-orbital overlaps, which offer charge transport channels, are mainly formed between the terminal groups of two adjacent A–D-A molecules^26,27^. In addition to considering the phase separation, domain size and purity in active layers, enhancing the intermolecular π-π stacking morphology is crucial to the facilitation of charge transport for A–D–A acceptors. Presently, the morphology optimization of NF-based blend films empirically follows the methods originally developed in fullerene-based blends, and both the advantages and disadvantages of the latter have been inherited. Considering the unique intermolecular packing features of the A–D–A acceptors, exploring a method that optimizes blend morphology while maintaining good device stability and reproducibility is crucial for further development of OSCs.

Herein, we present a method of directly enhancing the intermolecular π–π interaction by introducing volatilizable solid additives (SAs) in NF OSCs. We synthesize SA-1 with a similar chemical structure to that of the end-groups of an A–D–A acceptor IT-4F^25^. A film casted from the solution of equimolar IT-4F and SA-1, in which SA-1 volatilizes after thermal annealing (TA) at 140 °C, exhibits enhanced π–π stacking, leading to significantly improved electron mobility in comparison to the IT-4F film processed without SA-1. In OSC devices based on a fluorinated conjugated polymer (PBDB-TF) and IT-4F, the addition of SA-1 contributes to the optimization of the morphology of active layers, leading to the PCE increasing from 12.2 to 13.8%, which is comparable to the results using DIO as an additive^28^. More attractively, the devices processed with SA-1 show good tolerance to thickness variation of the active layer and exhibited good reproducibility and stability. We propose a working mechanism for this kind of additives by applying seven analogues of SA-1 with different volatility in NF OSCs. Furthermore, we have shown that the application of SA-1 can effectively improve the photovoltaic performance of NF OSCs based on different active layers.

## Results

### Design, synthesis and characterization of the solid additives

**Fig. 1:**
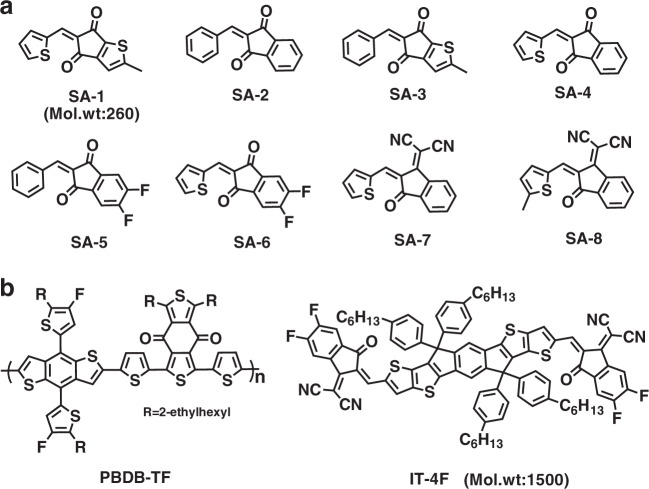
Chemical structures. **a** Chemical structures of eight designed solid additives, SA-x. **b** Chemical structures of the polymer donor PBDB-TF and the acceptor IT-4F

As illustrated in Fig. 1a, we designed eight SA-x (*x* = 1 to 8) to have similar chemical structures to those of the end-groups of IT-4F, expecting that the SA-x had good miscibility with IT-4F molecules. The SA-x can be easily synthesized by some low-cost compounds through the Knoevenagel condensation reaction (Supplementary Note 1) and have good solubility in commonly used solvents such as dichloromethane, chloroform and chlorobenzene. We measured the fundamental physicochemical properties of the SA-x by taking SA-1 as an example. As illustrated in Supplementary Fig. 1, in chloroform solution, SA-1 exhibits an absorption peak at 370 nm with an extinction coefficient of 3.1 × 10^4^ M^−1^ cm^−1^. The molecular configurations and molecular energy levels of SA-1 are calculated by density functional theory (DFT) and provided in Supplementary Figs 2 and 3.

### Application of SA-1 as an SA in OSCs

OSC devices were fabricated using a conventional device structure of indium tin oxide/poly(3,4-ethylenedioxythiophene):poly(styrenesulfonate)/PBDB-TF^29^: IT-4F/poly[(9,9-bis(3′-((*N*,*N*-dimethyl)-*N*-ethylammonium)-propyl)-2,7-fluorene)-*alt*-2,7-fluorene)-*alt*-2,7-(9,9-dioctylfluorene)]dibromide (PFN-Br)^30^/Al, where the PBDB-TF and IT-4F (their molecular structures are illustrated in Fig. 1b) work as a donor and an acceptor, respectively. Different amounts of SA-1 were added into the solution of PBDB-TF:IT-4F before film coating. Thermal annealing of the active film at 140 °C for 10 min was performed after the film coating. Detailed experimental procedures are provided in the methods section.

**Fig. 2:**
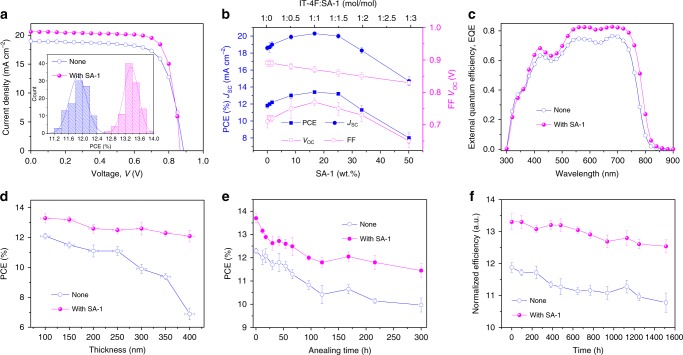
Device performance. **a** *J*−*V* curves and the histogram of PCE for PBDB-TF:IT-4F-based devices with/without SA-1. Thermal annealing of the active films at 140 °C for 10 min was performed. **b** Device parameters of PBDB-TF:IT-4F devices as a function of the mole ratios of IT-4F:SA-1 or the weight ratio of SA-1 incorporated into the casting solution (error bars show standard deviation from the mean). **c** EQE curves of the corresponding devices. **d** PCEs of the devices based on PBDB-TF:IT-4F processed with or without SA-1 under various active layer thicknesses (the horizontal error bars represent the standard deviation of the thicknesses and the vertical error bars show standard deviation from the mean). **e**, the thermal stability of OSC devices processed with or without SA-1 (error bars show standard deviation from the mean). **f** The long-term storage stability of OSC devices processed with or without SA-1 (error bars show standard deviation from the mean)

We first fabricated the PBDB-TF:IT-4F-based device processed without any additive, which exhibits a maximum PCE of 12.2% with an open-circuit voltage (*V*_oc_) of 0.89 V, a short-circuit current density (*J*_sc_) of 19.0 mA cm^−2^, and a fill factor (FF) of 0.72 (Fig. 2a and Table 1). When the mole ratio of IT-4F:SA-1 was drastically changed from 1:0.05 to 1:3, as shown in Fig. 2b and summarized in Supplementary Table 1, both the *J*_sc_ and FF first rose and then descended, whereas the *V*_oc_ decreases with an increase in SA-1 content and the corresponding *J*−*V* and EQE curves are plotted in Supplementary Fig. 4. As a result, the optimal device was obtained when 17.3 wt.% SA-1 was used with respect to IT-4F (the mole ratio of IT-4F:SA-1 was 1:1); it had a *J*_sc_ of 20.6 mA cm^−2^, an FF of 0.77 and a *V*_oc_ of 0.87 *V*, leading to a very good PCE of 13.8%. It should be noted that all the devices had over 13% PCEs when the weight ratio of SA-1 ranged from 8 to 25 wt.%. Fig. 2c displays the external quantum efficiency (EQE) curves of the device processed without additives and the optimal device. It is very clear that the addition of SA-1 leads to the enhancement of EQE values in the region of 400–800 nm, especially in 500–750 nm, thereby contributing to the improved *J*_sc_. The calculated integral current densities are 18.7 and 20.1 mA cm^−2^ for the devices processed with or without SA-1, respectively; these results are in good agreement with the *J*_sc_ values obtained from the *J*−*V* measurements.

**Table 1:** The effect of SA-1 on the photovoltaic parameters of devices based on different active layers

Active layer	SA-1^a^ (wt.%)	*V*_oc_ (V)	*J*_sc_ (mA cm^−2^)	FF	PCE (%)^b^
PBDB-TF:IT-4F	None	0.89 ± 0.01	18.8 ± 0.3	0.71 ± 0.02	11.9 ± 0.2 (12.2)
	17.3%	0.86 ± 0.01	20.2 ± 0.3	0.76 ± 0.01	13.3 ± 0.3 (13.8)
PBDB-TCl:IT-4F	None	0.90 ± 0.01	19.0 ± 0.4	0.71 ± 0.02	12.3 ± 0.3 (12.8)
	17.3%	0.86 ± 0.01	21.1 ± 0.2	0.75 ± 0.02	13.7 ± 0.4 (14.2)
PBDB-TF:IT-2F	None	0.94 ± 0.01	18.2 ± 0.2	0.72 ± 0.02	12.3 ± 0.3 (12.7)
	17.8%	0.92 ± 0.01	19.2 ± 0.3	0.76 ± 0.02	13.3 ± 0.3 (13.7)
PBTA-TF:IT-M	None	0.95 ± 0.01	18.2 ± 0.2	0.66 ± 0.03	11.4 ± 0.2 (11.8)
	17.5%	0.94 ± 0.01	19.0 ± 0.3	0.70 ± 0.01	12.4 ± 0.3 (12.8)
PBDB-T:ITIC	None	0.89 ± 0.01	16.2 ± 0.4	0.63 ± 0.03	9.1 ± 0.2 (9.4)
	17.8%	0.87 ± 0.01	17.1 ± 0.2	0.69 ± 0.02	10.2 ± 0.1 (10.4)
PBDB-T:ITCC	None	0.98 ± 0.01	15.2 ± 0.3	0.63 ± 0.02	9.3 ± 0.3 (9.7)
	18.2%	0.97 ± 0.01	15.6 ± 0.2	0.70 ± 0.01	10.2 ± 0.2 (10.6)
J52:IEICO	None	0.86 ± 0.01	13.4 ± 0.2	0.51 ± 0.02	5.9 ± 0.2 (6.2)
	14.9%	0.86 ± 0.01	14.5 ± 0.4	0.57 ± 0.02	6.7 ± 0.4 (7.3)

^a^The weight ratios of SA-1 for different systems are calculated from each case when the mole ratio of NF acceptor and SA-1 is 1:1

^b^The maximum PCEs are shown in the parentheses

Then, we fabricated the optimal OSCs with active layer thicknesses varying from 100 to 400 nm and studied the impact of the SA-1 on the device stability. As illustrated in Fig. 2d and Supplementary Table 2, when the thickness increased to approximately 400 nm, the PCEs of OSCs fabricated without using SA-1 sharply dropped to 6.9% while PCEs above 12% remained for the OSCs fabricated by including SA-1, which will benefit the large-scale fabrication of OSCs through roll-to-roll printing technology^12,31,32^. Furthermore, we investigated the long-term thermal stability of the optimal OSCs. As demonstrated in Fig. 2e, after 300 h of annealing at 140 °C, the devices fabricated with SA-1 yielded PCEs of around 11.5%, while those processed without SA-1 had PCEs lower than 10%. We also studied the long-term storage stability of the devices (encapsulated in a N_2_-filled glovebox), and PCEs of 12.5% were maintained after storage for 1500 h (Fig. 2f). These results suggest that the use of SA-1 in the active layer not only enhances the device’s photovoltaic performance but also provides benefits of good long-term thermal and storage stability.

To clarify the effect of TA, we fabricated the as-cast PBDB-TF:IT-4F and PBDB-TF:IT-4F+SA-1 devices for comparison. As demonstrated in Supplementary Fig. 5, the as-cast device exhibits a PCE of 11.6%, a *J*_sc_ of 18.7 mA cm^−2^ and an FF of 0.69. When 17.3 wt.% SA-1 was added, the PBDB-TF:IT-4F-based device without TA shows a lower PCE of 11.1% with a *J*_sc_ of 20.2 mA cm^−2^ and an FF of 0.61. The photovoltaic parameters of SA-1 processed devices under different TA temperatures were also investigated (Supplementary Table 4).

We ascribed the simultaneous enhancement of *J*_sc_ and FF in OSCs to the improved charge transport properties in the blend films processed with SA-1. Therefore, we first measured the electron mobilities of blend films through the space charge limited current (SCLC) method^33^ with an electron-only device structure. As showed in Supplementary Fig. 6a and summarized in Supplementary Table 5, the electron mobility of the IT-4F+SA-1/TA film is 1.4 × 10^−3^ cm^2^ V^−1^ s^−1^, which is approximately one order of magnitude higher than that of the IT-4F/TA film (2.0 × 10^−4^ cm^2^ V^−1^ s^−1^). The electron mobilities of the PBDB-TF:IT-4F blend films with different treatments were also measured. The electron mobility of as-cast film shows no obvious improvement after adding SA-1, while the electron mobility of SA-1-added film was improved from 1.4 × 10^−4^ to 1.3 × 10^−3^ cm^2^ V^−1^ s^−1^ after TA. Photo-induced charge-carrier extraction in linearly increasing voltage (photo-CELIV) measurements were also conducted^34^. The photo-CELIV mobility (Supplementary Fig. 6d) of PBDB-TF:IT-4F+SA-1/TA film (1.2 × 10^−4^ cm^2^ V^−1^ s^−1^) is also higher than both the PBDB-TF:IT-4F+SA-1 film (6.8 × 10^−5^ cm^2^ V^−1^ s^−1^) and the PBDB-TF:IT-4F/TA film (7.6 × 10^−5^ cm^2^ V^−1^ s^−1^). Furthermore, compared with those of the device processed without SA-1, the bimolecular recombination was more suppressed in the SA-1-processed device (Supplementary Fig. 7).

### Influence of SAs on blend morphology

**Fig. 3:**
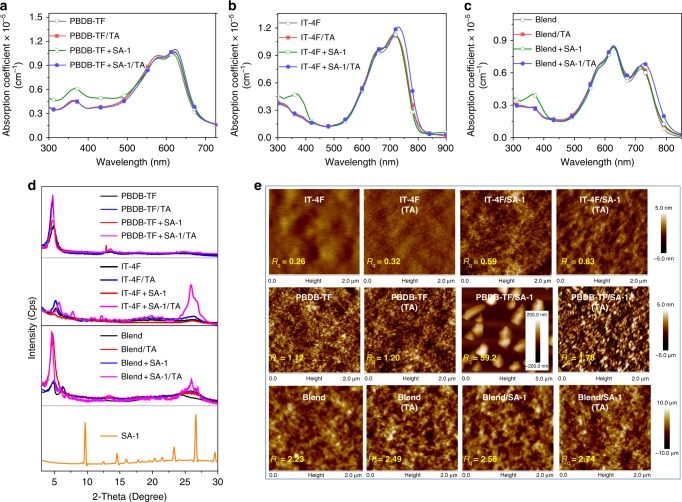
The influence of SA-1 on the intermolecular interaction and the film morphology. Absorption spectra with absorption coefficients of (**a**) PBDB-TF, PBDB-TF/TA, PBDB-TA+SA-1, and PBDB-TF+SA-1/TA films; **b** IT-4F, IT-4F/TA, IT-4F+SA-1, and IT-4F+SA-1/TA films; **c** blend, blend/TA, blend+SA-1, and blend+SA-1/TA films. **d** The corresponding X-ray diffraction patterns and that of SA-1 film without TA. **e** The corresponding AFM height images  (A specified scale bar is used for the image of PBDB-TF+SA-1 film)

As is known, the optical properties of organic semiconductors have a strong connection with the aggregation behavior in solid films^35,36^. First, as depicted in Fig. 3a, the UV-vis absorption spectra of the films at different conditions were measured. When comparing the SA-1-added and the SA-1-added/TA films, the characteristic absorption peaks of SA-1 in shortwave region vanish after TA. For the polymer PBDB-TF/TA film, the use of SA-1 affects the absorption coefficient slightly. For the IT-4F+SA-1/TA film, the absorption peak red-shifts about 14 nm with increased absorption coefficient approximately 7% when compared with the as-cast IT-4F film, which should be ascribed to the enhanced intermolecular π–π interaction of IT-4F. Detailed optical properties of PBDB-TF, IT-4F and blend films under different treatments are summarized in Supplementary Table 6. As exhibited in Fig. 3b, a similar phenomenon can be observed in the PBDB-TF:IT-4F-based blend film, *i*.*e*. that SA-1 has the obvious effect of enhancing intermolecular π*–*π interaction on IT-4F rather than the polymer donor.

Then, we measured the X-ray diffraction (XRD) patterns of the donor, acceptor, and the blend films processed with different conditions. As indicated in Fig. 3c, the diffraction peaks of the SA-1 film made through the solution process are sharp and intense, indicating that SA-1 has high crystallinity. The annealed IT-4F film processed without SA-1 (blue line) exhibits two weak diffraction peaks at 2-Theta of 5.5° and 26°, corresponding to a *d*_100_-spacing value of 15.9 Å and a *d*_010_-spacing value of 3.4 Å, which are stronger than those of the as-cast IT-4F film (black line). By adding an equimolar ratio of SA-1 into the solution for making the IT-4F film, the characteristic diffraction peaks of SA-1 cannot be distinguished, suggesting that it is well miscible with IT-4F. Interestingly, the *d*_010_ peak of IT-4F, which should be ascribed to intermolecular π–π interaction, can be significantly enhanced in the IT-4F+SA-1/TA film. For the blend film, the use of SA-1 also enhances the (010) diffraction peak of IT-4F. For the PBDB-TF/TA film, the use of SA-1 has a litter influence on its crystallinity. For the blend/TA film, it shows an enhanced (010) peak by using SA-1. To further study the crystallinity of the films, we carried out the 2D GIWAXS measurements. The 2D GIWAXS patterns and the corresponding intensity profiles in the out-of-plane (OOP) and in-plane directions are provided in Supplementary Fig. 8. For the IT-4F-based films with different treatments, the IT-4F+SA-1/TA film shows a pronounced (010) peak in the OOP direction, suggesting more ordered molecular stacking.

We then investigated the effect of SA-1 on phase separation morphology in the surfaces of the films using atomic force microscopy (AFM). The as-cast films processed without SA-1 show smooth and uniform surfaces (Fig. 3d). The corresponding mean-square surface roughness (*R*_q_) values are slightly increased after TA at 140 °C for 10 min. The as-cast film of the equimolar mixture of IT-4F and SA-1 has a smooth surface, although the neat SA-1 has a very poor film formation property. When the content of SA-1 further increases (IT-4F:SA-1, mol:mol = 1:2 or 1:3), some tiny aggregations of SA-1 appear, while most of the film is still quite smooth (Supplementary Fig. 9). In the as-cast PBDB-TF+SA-1 film (wt/wt = 100:17.3), SA-1 forms large crystals with a high *R*_q_ of 59.2 nm, but the film still shows normal morphology in the zoom without crystals. For the as-cast PBDB-TF:IT-4F blend film, the nanoscale phase-separation morphology can be maintained after adding the same content of SA-1, which could be ascribed to the good miscibility of SA-1 and IT-4F. After TA, the *R*_q_ of the IT-4F+SA-1/TA film is 0.83 nm, which is higher than that of the IT-4F/TA film (0.32 nm). The large crystals of SA-1 on the surface of PBDB-TF films disappeared with a slightly higher *R*_q_ than the as-cast PBDB-TF film observed. From the transmission electron microscopy (TEM) image, there is no any hollow observed for the blend+SA-1/TA film. We find the film thickness dropped approximately 10 nm after treated with TA (Supplementary Fig. 10), suggesting more condensed molecular arrangement in the blend.

### Proposed working mechanism of SAs

**Fig. 4:**
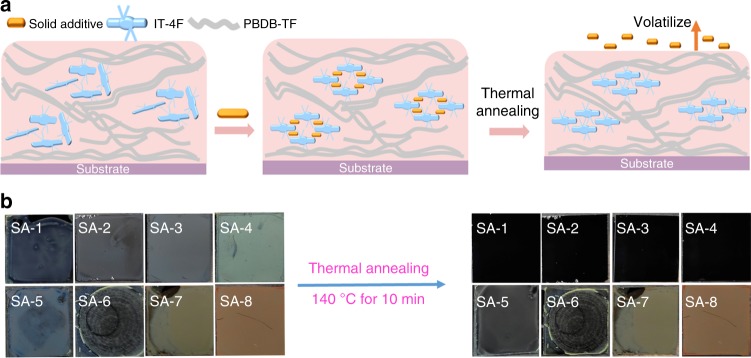
Proposed mechanism of volatilizable SAs. **a** Schematic diagram of working mechanism of SAs. **b** Photographs of spin-coated films of eight SAs. Then the films were thermal annealed at 140 °C for 10 min

Based on the photovoltaic characterizations, absorption spectra, as well as XRD and AFM results, we speculate the role of the SA-1 on affecting photovoltaic properties of the PBDB-TF:IT-4F blend as demonstrated in Fig. 4a. During the spin-coating process, SA-1 can be well mixed with IT-4F and they may act as small bridges to enhance the π-stacking between the two IT-4F molecules. During the TA process, SA-1 is removed from the film, leaving more room for the self-assembly of IT-4F and forming a more condensed and ordered molecular arrangement, which makes it possible for a strong π–π interaction among IT-4F molecules. As a result, even if the *V*_oc_ is slightly reduced caused by the changes of morphologies, the photovoltaic properties of the blend film can be significantly improved by using SA-1 as the additive. In this process, the donor matrix acts as a limit to the excessive movement of IT-4F molecules, which helps to maintain the good bi-continuous interpenetrating networks. To verify the proposed mechanism, we first studied the volatility of SA-1 by the detailed thermogravimetric analysis (TGA) and solution absorption before and after its volatilization. Then, we applied SA-1 in other efficient NF OSCs and investigated the volatility of the other seven small molecular materials (SA-x, *x* = 2 to 8) as illustrated in Fig. 1a and used them as solid additive for device fabrication, respectively.

First, we measured the TGA at different conditions. As shown in the Supplementary Fig. 11a, we changed the heating rates of 1, 10 and 20 °C min^−1^ and SA-1 shows different *T*_d_ (5% weight loss) of 209, 241 and 263 °C, and the weight loss starts from around 180 °C. We think the volatilization of SA-1 is a slow process, therefore, we hold the heating temperature of 140, 150, 160 and 180 °C for 2 h. We can find a clear weight loss at 140 °C, implying the SA-1 have volatility at 140 °C. The increasing temperature will accelerate the volatilization of SA-1 significantly. After the measurement, we found the SA-1 on the top of crucible, suggesting it is not decomposed. We then put the SA-1 powder and made a thin SA-1 film (about 40 nm) on the silicon substrates and observed their changes under thermal annealing. We found that the SA-1 film gradually changed over time and totally vanished 2 min later, and the substrate became clean (Supplementary Fig. 11e). However, the SA-1 powder showed really little change. The AFM images and photos of SA-1 film with or without TA are shown in Supplementary Fig. 12. Furthermore, we measured the solution absorption spectra of PBDB-TF:IT-4F blend with different weight ratio of SA-1 and the solution of chloroform-dissolved films under different conditions to confirm that SA-1 could leave totally from the active layer after TA at 140 °C (Supplementary Fig. 13). Furthermore, Fourier transform infrared (FT-IR) spectra was also applied to confirm the volatilization of SA-1 from the blend film (Supplementary Fig. 14).

To further verify this mechanism, we applied the SA-1 in other efficient NF OSCs by using different polymers and A–D-A acceptors, including PBDB-TCl:IT-4F, PBTA-TF:IT-M, PBDB-TF:IT-2F, PBDB-T:ITIC, PBDB-T:ITCC and J52:IEICO (the chemical structures are shown in Supplementary Fig. 15). The *J* − *V* curves of these devices processed with or without SA-1 are provided in Supplementary Fig. 16, and the detailed photovoltaic parameters are summarized in Table 1. Clearly, the addition of SA-1 in different systems can simultaneously enhance *J*_sc_ and FF values of the devices; this is very similar to the phenomenon observed in PBDB-TF:IT-4F-based devices. Impressively, a maximum PCE of 14.2% was recorded for the PBDB-TCl:IT-4F-based OSCs, which is comparable to the result obtained by using DIO as a solvent additive in our recent work^37^.

As illustrated in Fig. 4b, these small molecular materials (SA-x) possess different volatilities (Supplementary Figs 17 and 18) in the spin-coated solid films. For instance, after TA at 140 °C for 10 min, the neat films of SA-1, SA-2, SA-3 and SA-4 can be completely removed while many residues remained in the films of SA-5 and SA-6. For the SA-7 and SA-8 films, there was no obvious change after TA. As presented in Table 2 and Supplementary Fig. 19, the PBDB-TF:IT-4F-based OSC devices fabricated with SA-2, SA-3 or SA-4 exhibit good PCE around 13.6%, which is comparable to that of the device fabricated with SA-1. However, the photovoltaic properties of the devices with the other four additives are obviously lower, especially for the devices processed with SA-7 and SA-8. We also found that the film thickness of PBDB-TF:IT-4F blend processed with SA-2, SA-3 or SA-4 decreased approximately 10 nm after TA, which is similar to that observed in the blend film processed with SA-1, while the thicknesses of the blend film processed with other four additives were almost unchanged after TA. Therefore, we can conclude that SA-1, SA-2, SA-3 and SA-4 exhibit similar functions for improving photovoltaic performance of the PBDB-TF:IT-4F-based OSCs, whereas the other four materials are not appropriate because of their weaker volatilities.

**Table 2:** Photovoltaic parameters for PBDB-TF:IT-4F based devices with different SAs incorporated into the casting solution

Additive	Mol.wt.	wt.%^a^	*V*_oc_ (V)	*J*_sc_ (mA cm^−2^)	FF	PCE (%)^b^
SA-2	234	15.6	0.87 ± 0.01	20.4 ± 0.3	0.75 ± 0.01	13.3 ± 0.2 (13.6)
SA-3	260	17.3	0.87 ± 0.01	20.3 ± 0.2	0.75 ± 0.02	13.2 ± 0.3 (13.5)
SA-4	254	16.0	0.87 ± 0.01	20.2 ± 0.2	0.75 ± 0.02	13.2 ± 0.3 (13.6)
SA-5	270	18.0	0.88 ± 0.01	20.2 ± 0.2	0.71 ± 0.01	12.6 ± 0.2 (12.9)
SA-6	276	18.4	0.88 ± 0.01	19.9 ± 0.3	0.70 ± 0.02	12.2 ± 0.3 (12.6)
SA-7	288	19.2	0.85 ± 0.01	19.8 ± 0.4	0.68 ± 0.02	11.4 ± 0.2 (11.6)
SA-8	302	20.1	0.87 ± 0.01	18.9 ± 0.4	0.56 ± 0.04	9.3 ± 0.5 (10.0)

^a^The weight ratios of different additives for PBDB-TF:IT-4F-based devices were calculated from each case when the mole ratio of IT-4F: the additive was 1:1

^b^The maximum PCEs are shown in the parentheses

### The superiority of SAs compared with solvent additives

**Fig. 5:**
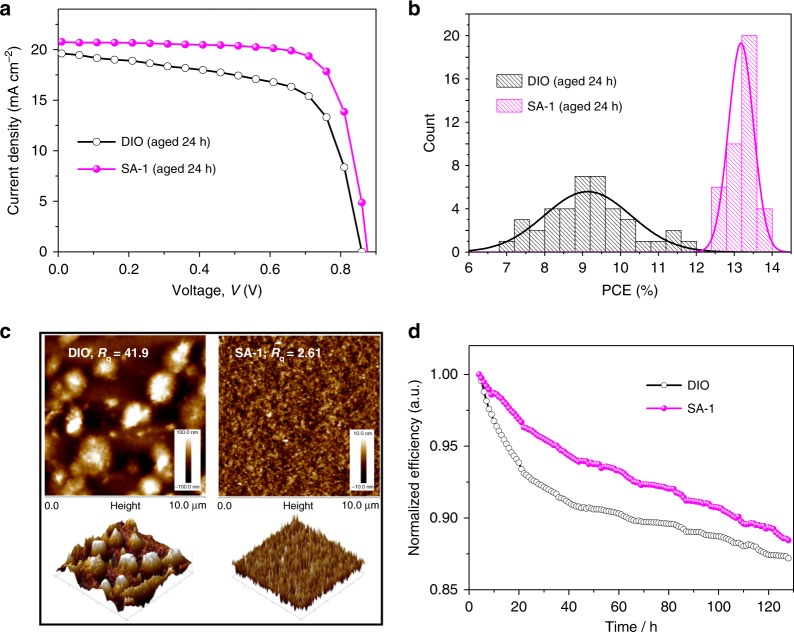
The advantages of SAs over solvent additives. **a** *J* − *V* curves of OSCs fabricated by aged films using DIO or SA-1. **b** The histogram of PCE for aged devices with DIO or SA-1. **c** AFM height image and 3D images of PBDB-TF:IT-4F films with DIO or SA-1, for which the casted active layer was annealed after 24 h standing. **d** Photo-stability of PBDB-TF:IT-4F-based devices with DIO or SA-1 (encapsulated in air, AM 1.5 radiation to illumination of 100 mW cm^−2^ for 130 h)

We fabricated the PBDB-TF:IT-4F-based device using the most widely used solvent additive DIO. As presented in Supplementary Table 7, the devices using DIO as additive followed by TA can achieve comparable PCE to that of the SA-1-processed OSCs. However, as illustrated in Fig. 5a, b, when the active layer films have been treated with 24 h-standing before TA, the devices fabricated using DIO exhibit lower PCE (9.3 ± 1.3%) and poor reproducibility. From the AFM images in Fig. 5c, we can see that the aged film processed with DIO exhibits large aggregations with an *R*_q_ of 41.9 nm, due to the slow evaporation of residual DIO in the storage period. By contrast, the aged film with SA-1 still exhibits smooth and uniform morphology, and the resulting devices fabricated using SA-1 still have good device performance (PCE of 13.2 ± 0.3%). Benefiting from the intrinsic nature of solid additive, the OSCs processed with SA-1 exhibit better morphological stability than those with residual DIO, and the former contribute to higher yield and reproducibility in large-scale production.

Additionally, we investigated the stability of the optimal OSCs fabricated with SA-1 or DIO under illumination in air. After illumination for 130 h (encapsulated, AM 1.5 radiation to illumination of 100 mW cm^−2^), the device fabricated with SA-1 delivered a PCE of 12.0% and showed better stability than the device with DIO (Fig. 5d).

According to the literature, the photovoltaic performance of OSCs processed with DIO is greatly affected by the volume of addition, which is typically below 5% or even 0.5% (v/v) and needs be carefully controlled^6,20–22^. However, as mentioned above, the devices can maintain their good performances when the content of SA-1 varies in a large range, from 8 to 25 wt.%. These advantages of volatilizable SAs, including better morphological stability, illumination stability and insensitivity to the amount of additive, will be beneficial for the future industrial application of OSCs.

## Discussion

In conclusion, we report a method of applying volatilizable SAs to enhance photovoltaic performance of NF OSCs based on A–D–A-type acceptors. By considering the molecular structure of the NF acceptor IT-4F, we designed and synthesized a volatilizable solid additive SA-1, which can be well mixed with IT-4F and volatilized by TA. The intermolecular π–π interaction and charge transport properties of IT-4F can be significantly enhanced since the more condensed and ordered molecular arrangement after the SA-1 volatilized from the active layer, which contributes to the enhancement of *J*_sc_ and FF of the optimal OSCs. Furthermore, the device fabricated by using SA-1 possesses better device stability and reproducibility than the most widely used solvent additive, DIO. We also demonstrate that some of the SA-x, which have similar volatility to that of SA-1, can also be effective for achieving high-efficient devices, whereas SA-1 can be a general additive in varied active layers for improving the photovoltaic performance of NF OSCs. Our results suggest that using volatilizable SAs is an alternative and potential method of making OSCs more competitive in future industrialization and that designing specific additives for different active materials will be a promising direction in the photovoltaic field.

## Methods

### Device fabrication and testing

The conventional OSC devices were fabricated and characterized by using a device structure of ITO/PEDOT:PSS)/active layer/PFN-Br/Al, where PEDOT:PSS and PFN-Br served as the hole-transport layer and electron-transport layer, respectively. The cleaned ITO glasses were treated by UV-ozone for 15 min before about 20 nm thick of PEDOT:PSS was spin-coated. Then the ITO substrate were placed in an oven for 15 min at 150 °C. The active layer, PBDB-TF:IT-4F blend was dissolved in chlorobenzene (CB) solvent at a polymer weight concentration of 11 mg mL^−1^, and the donor: acceptor ratio was kept at 1:1. SA-x of the same concentration was also dissolved in CB. After adding SA-x, the blend solution was stirred for more than 20 min. After the blend solution were spin-coated onto ITO glasses, TA for 10 min at 140 °C was utilized to optimize the morphology of active layer. Then the electron-transport layer of PFN-Br (about 5 nm) was coated on the top of active layer at 3000 rpm for 30 s. Finally, about 90 nm of Al with an area of 4.0 mm^2^ were deposited onto the active layer under high vacuum. The preparation of the other devices based on PBDB-TCl:IT-4F, PBTA-TF:IT-M, PBDB-TF:IT-2F, PBDB-T:ITIC, PBDB-T:ITCC and J52:IEICO were the same as the procedures discussed above. Except for the spin-coating of PEDOT:PSS, the other the processes were all carried out in an nitrogen-filled glovebox. The *J−V* measurement was performed via the solar simulator (SS-F5-3A, Enlitech) along with AM 1.5 G spectra whose intensity was calibrated by the certified standard silicon solar cell (SRC-2020, Enlitech) at 100 mW cm^−2^. The external quantum efficiency (EQE) data were obtained by using the solar-cell spectral-response measurement system (QE-R, Enlitech). The devices for examining the storage stability and light stability were fabricated by using a structure of ITO/ZnO/active layer/MoO_3_/Al. AM 1.5 G spectrum at 100 mW cm^−2^ along with a National Institute of Metrology, China calibrated reference cell was used to measure the OSCs. As shown in the Supplementary Fig. 20, the best OSC device was sent to the National Institute of Metrology, China (NIM), to make a certification by using an area of 3.78 mm^2^. The confirming PCE was 13.7% with a *V*_oc_ of 0.88 *V*, a *I*_sc_ of 0.77 mA and an FF of 0.765.

### Morphology characterization

AFM images were obtained by a Nanoscope *V* AFM on tapping mode. For the films with SA-1, 17.3 wt.% of SA-1 with respect to PBDB-TF or IT-4F were added in each blend solution before spin-coating. All the film samples with or w/o SA-1 were spin-coated from the respective solutions onto ITO-PEDOT:PSS substrate. X-ray diffraction (XRD) patterns were collected using a Rigaku D/max 2500 X-ray diffractometer and the samples were prepared on Si substrates by drop-casting. The GIWAXS data were obtained on a XEUSS SAXS/WAXS SYSTEM (XENOCS, FRANCE) at the National Center for Nanoscience and Technology (NCNST, Beijing).

### Synthesis of SA-1 and other solid additives

The detailed synthesis routes can be found in the Supplementary Notes 1 to 8. The NMR data, elemental and mass analysis of all the modulators are included as Supplementary Figs 21 to 44.

## Electronic supplementary material

Supplementary Informantion

## Data Availability

The data that support the findings of this study are available from the corresponding author upon reasonable request.
