# Supplementary material for: Design and application of volatilizable solid additives in non-fullerene organic solar cells
Source: Nat Commun. 2018 Nov 7;9:4645. doi: 10.1038/s41467-018-07017-z (PMC6220167; doi:10.1038/s41467-018-07017-z)
Supplement: Supplementary file 1 — Supplementary Informantion [file 41467_2018_7017_MOESM1_ESM.pdf]

SUPPLEMENTARY INFORMATION

Design and Application of Volatilizable Solid Additives in Non-Fullerene Organic Solar Cells

Yu et al.

Supplementary Figures

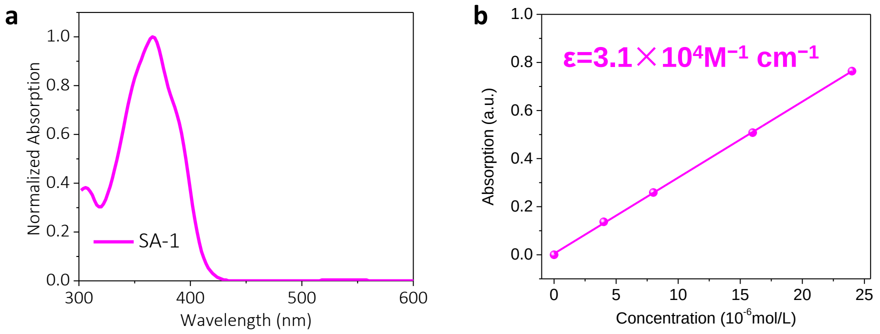

Supplementary Figure 1. **a**, Normalized absorption spectra and **b**, absorption coefficient of SA-1 in diluted CF solution.

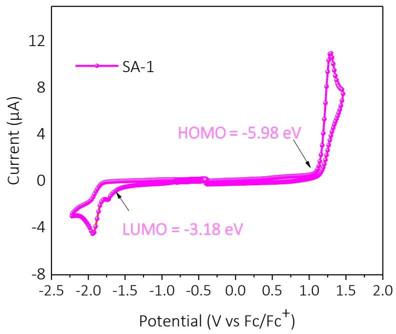

Supplementary Figure 2. Cyclic Voltammetry plots of SA-1 in dichloromethane solution (1 mM) with a scan rate of 50 mV s^{-1}. The highest occupied molecular orbital (HOMO) and lowest unoccupied molecular orbital (LUMO) levels are calculated as −5.98 and −3.18 eV, respectively

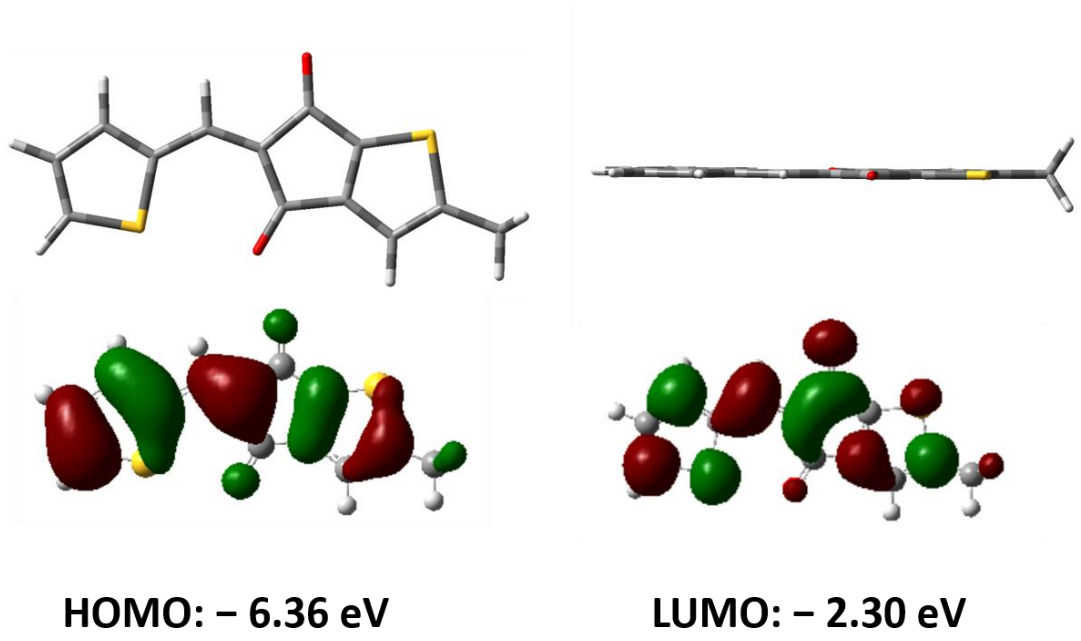

Supplementary Figure 3. Optimized molecular conformations and energy levels of SA-1 calculated by DFT-based theoretical calculations at the B3LYP/6-31G(d,p) level.

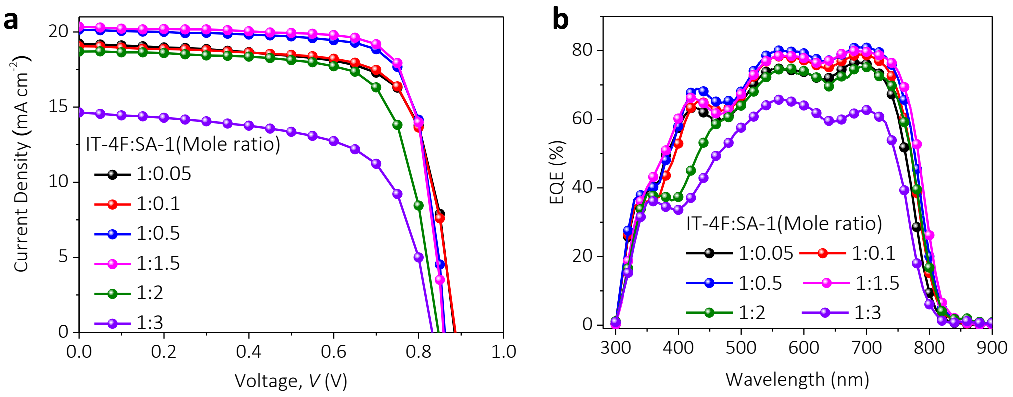

Supplementary Figure 4. a, *J*–*V* curves and **b**, EQE curves for PBDB-TF:IT-4F based devices with different mole ratios of IT-4F:SA-1.

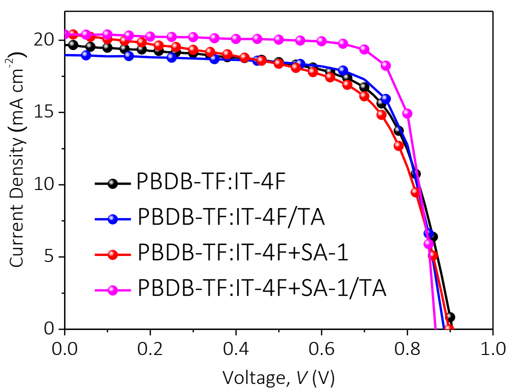

Supplementary Figure 5. *J–V* curves of the PBDB-TF:IT-4F based device with different treatments, including as-cast, annealed, SA-1 added as-cast and SA-1-added annealed devices.

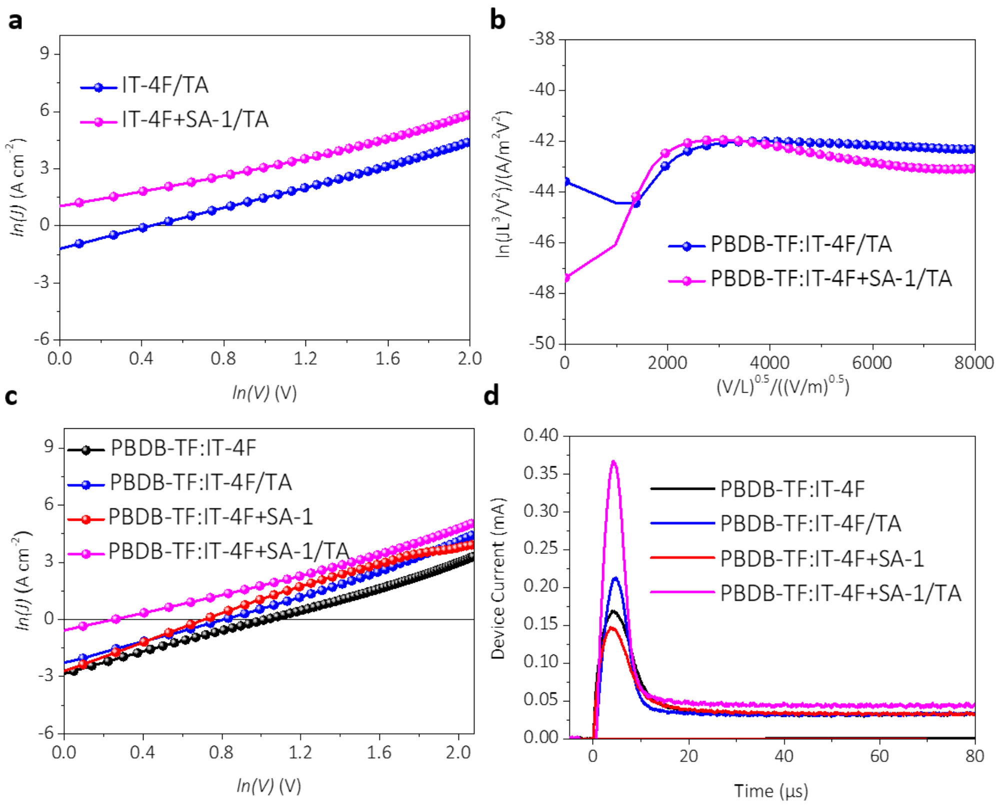

Supplementary Figure 6. a, Ln (J) versus Ln (V) plots of IT-4F/TA and IT4F+SA-1/TA films(Date has not been corrected for V_{bi}). **b**, Ln(JL^3/V^2) versus $(V/L)^{0.5}$ plots of the annealed blend films based on PBDB-TF:IT-4F processed with or w/o 16.9 wt.% of SA-1 for SCLC measurements. **c**, Ln(J) versus Ln(V) plot of PBDB-TF:IT-4F blend films with different treatments, including as-cast, TA, SA-1-added, and SA-1-added TA films. **d**, the photo-CELIV plots of the corresponding devices.

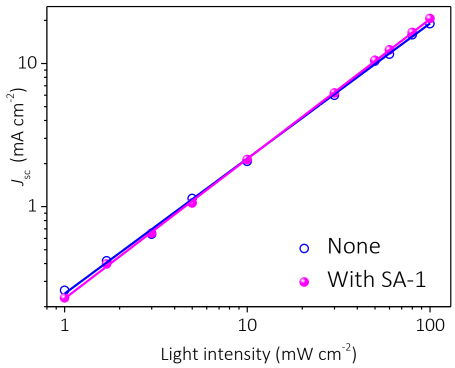

Supplementary Figure 7. J_{sc} versus light intensity of PBDB-TF:IT-4F based devices processed with or without SA-1 and the devices are treated with thermal annealing. The dependence of J_{sc} on the light density (P_{light}) was applied to investigate the charge recombination in the devices. The power-law exponent of relationship J_{sc}/P_{light} for the SA-1-processed device increase to 0.98 from 0.94 for that of the device processed w/o SA-1, which implies that the bimolecular recombination is more suppressed in SA-1-processed device.

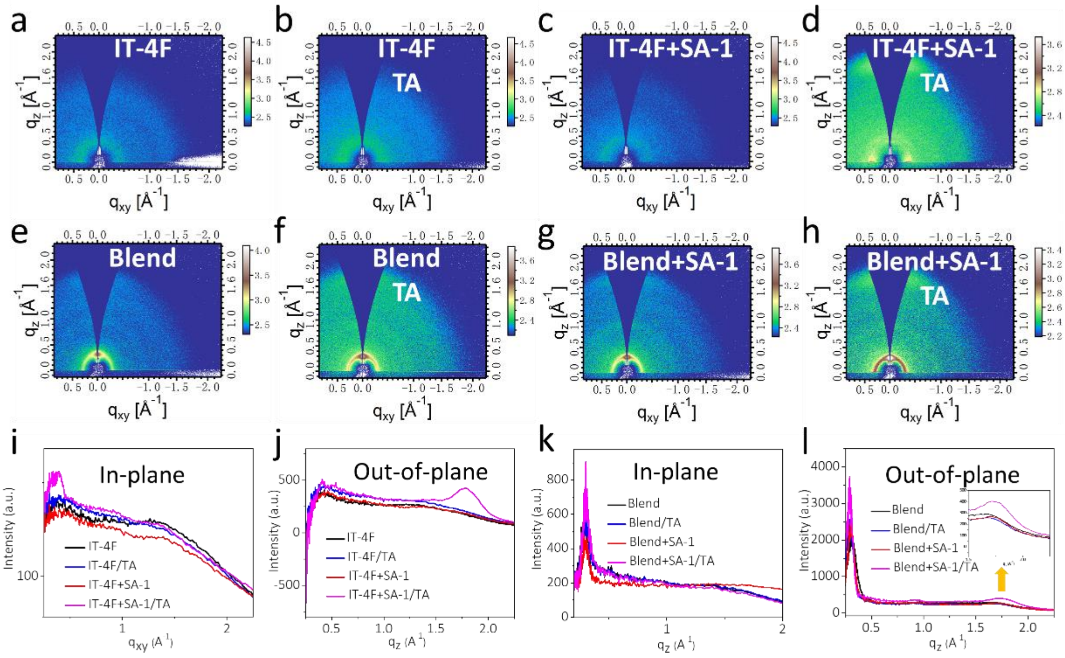

Supplementary Figure 8. a-h, 2D GIWAXS patterns of IT-4F and PBDB-TF:IT-4F blend film under different treatments, including as-cast film, processed with TA, SA-1-added as-cast film and the SA-1-added film processed with TA. **i,** In-plane and **j,** Out-of-plane cuts of the GIWAXS patterns for the IT-4F films with different treatments. **k,** In-plane and **l,** Out-of-plane cuts for the blend films.

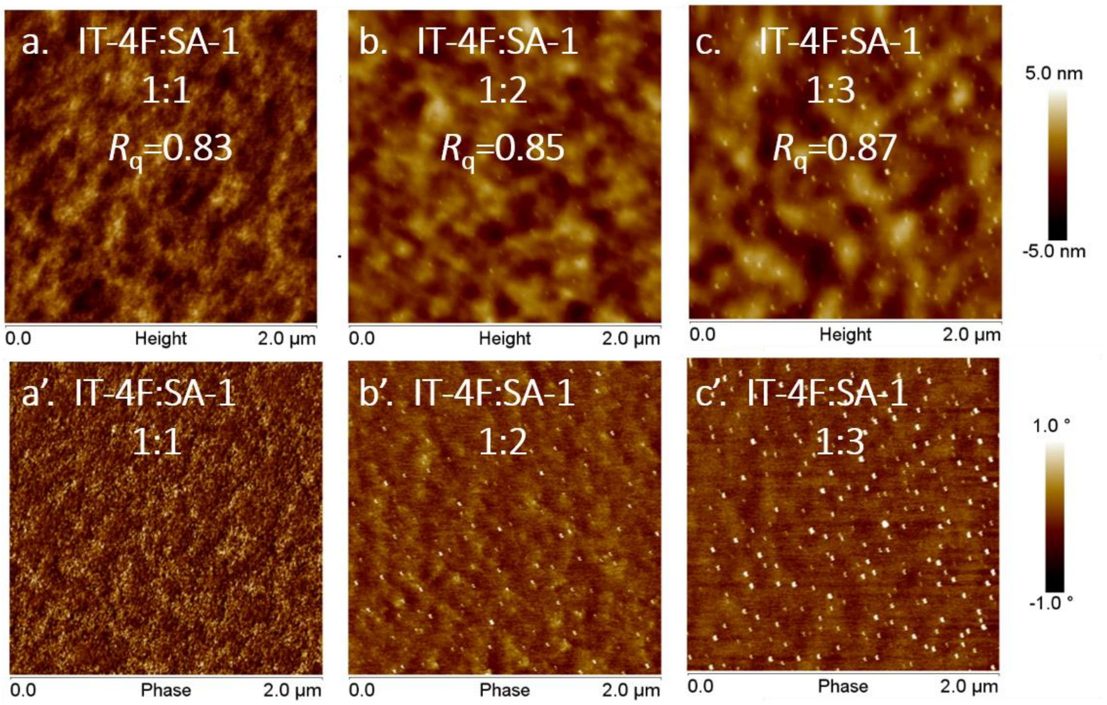

Supplementary Figure 9. AFM height and phase images of **a-a'**, SA-1 and the blend films based on IT-4F:SA-1 with varied mole ratios, including **b-b'**, 1:1, **c-c'**, 1:2, and **d-d'**, 1:3.

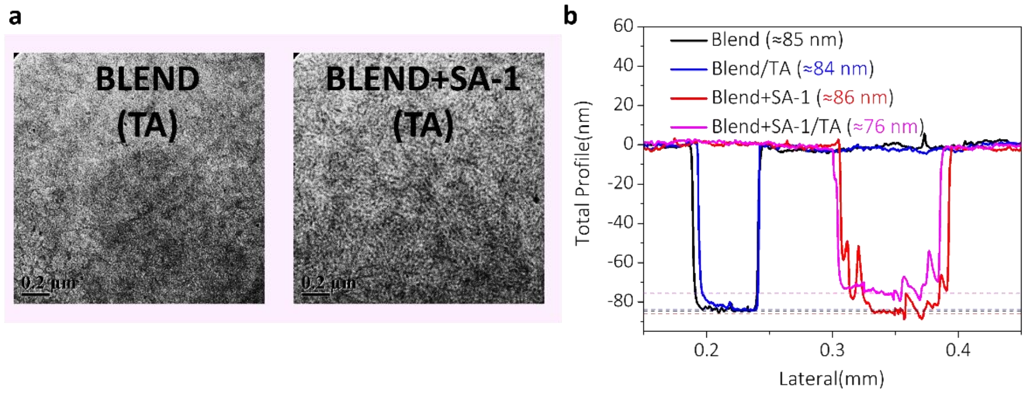

Supplementary Figure 10. a, TEM height and phase images of PBDB-TF:IT-4F/TA and PBDB-TF:IT-4F+SA-1/TA films. **b,** The film thickness of PBDB-TF:IT-4F blend films processed with different conditions. (We fabricated the blend and the blend+SA-1 films and the thicknesses of them are measured as 85 and 86 nm, respectively. After thermal annealing, we measured the in-situ thickness and found that the blend and blend/TA films showed similar film thickness, while comparing with the blend+SA-1 film, the blend +SA-1/TA film was reduced by about 10 nm.)

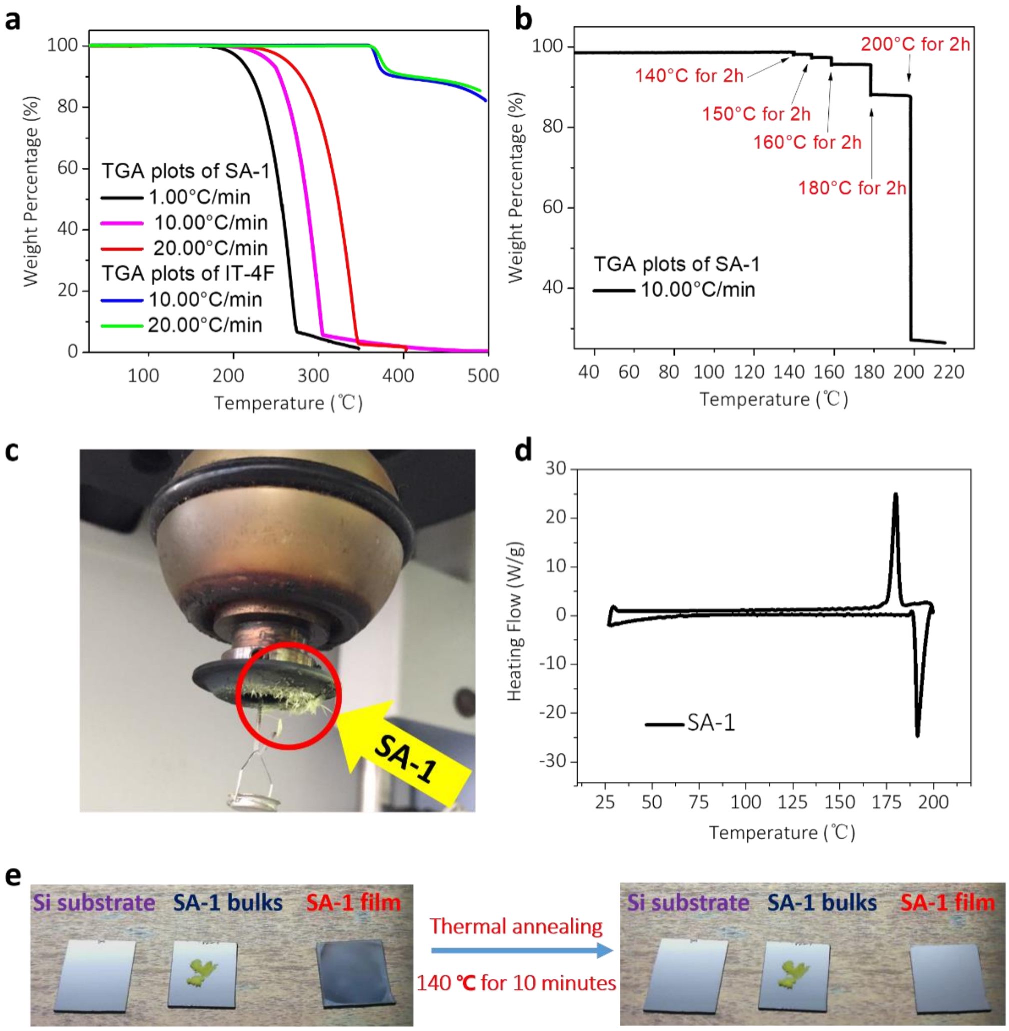

Supplementary Figure 11. a, Thermogravimetric Analysis (TGA) of SA-1 and IT-4F at different scan rates. **b**, TGA plot of SA-1 at a scan rat of 10.0 °C min^{-1} and in the heating process, the temperature was held for 2h at 140, 150, 160, 180 and 200 °C, respectively. **c**, Photo of the top of crucible after the heating and the cooling process of SA-1. **d**, Differential Scanning Calorimetry (DSC) curves of SA-1. **e,** Photos of SA-1 bulks and the respective spin-coated film on Si substrates. Then the films were thermal annealed at 140 °C for 10 minutes. Furthermore, a video recording the volatilization of SA-1 bulks and film is attached.

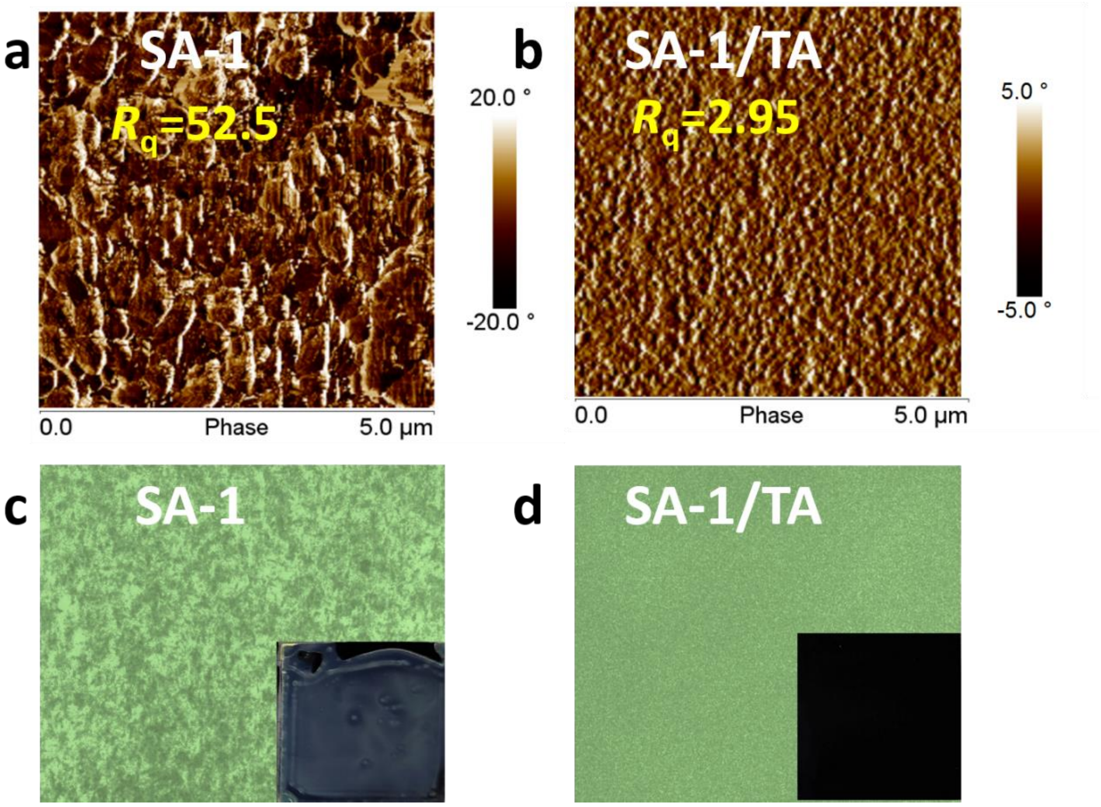

Supplementary Figure 12. **a,** AFM phase images of SA-1 film and b, the corresponding one after thermal annealing at 140°C for 10 minutes. **c-d,** Photos of SA-1 and SA-1/TA films on ITO substrate captured by the camera of AFM and the corresponding films on Fe substrate captured by camera.

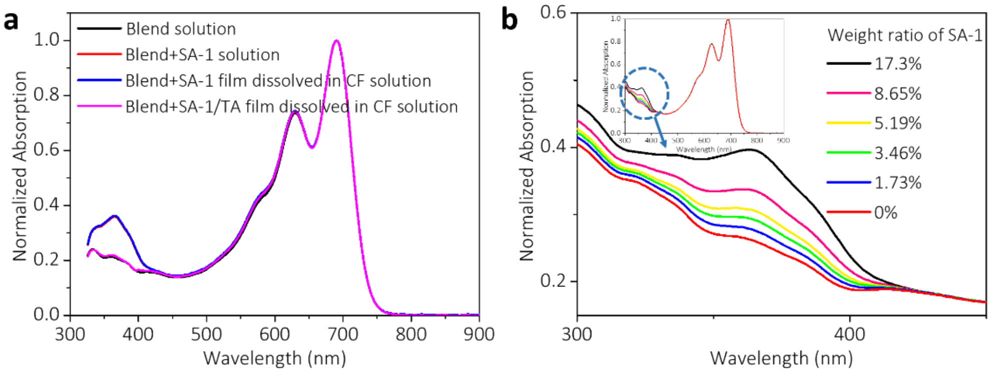

Supplementary Figure 13. a, Normalized absorption spectra of a, PBDB-TF:IT-4F blend solution, the solution of blend with 16.9 wt.% SA-1, the CF dissolved solution of blend+SA-1 film, and that of the blend+SA-1/TA film. **b,** Normalized absorption spectra of PBDB-TF:IT-4F solution blended with different weight ratio of SA-1 (the corresponding mole ratios of SA-1:IT-4F are 1:1, 0.5:1, 0.3:1, 0.2:1, 0.1:1 and 0:1, respectively). (The absorption peak in the region of 300-450 nm is sensitive to the amount of SA. For example, it is clear to see the absorption peak of SA-1, when blended with even less than 2 wt.% of SA-1 to the PBDB-TF:IT-4F solution. For the CF dissolved solution of blend+SA-1/TA film, its absorption spectra nearly overlaps with that of the blend solution without SA, suggesting that the content of SA in the blend is negligible. The results have proved that SA-1 totally escaped from the active layer film after thermal annealing at 140 °C for 10 min.).

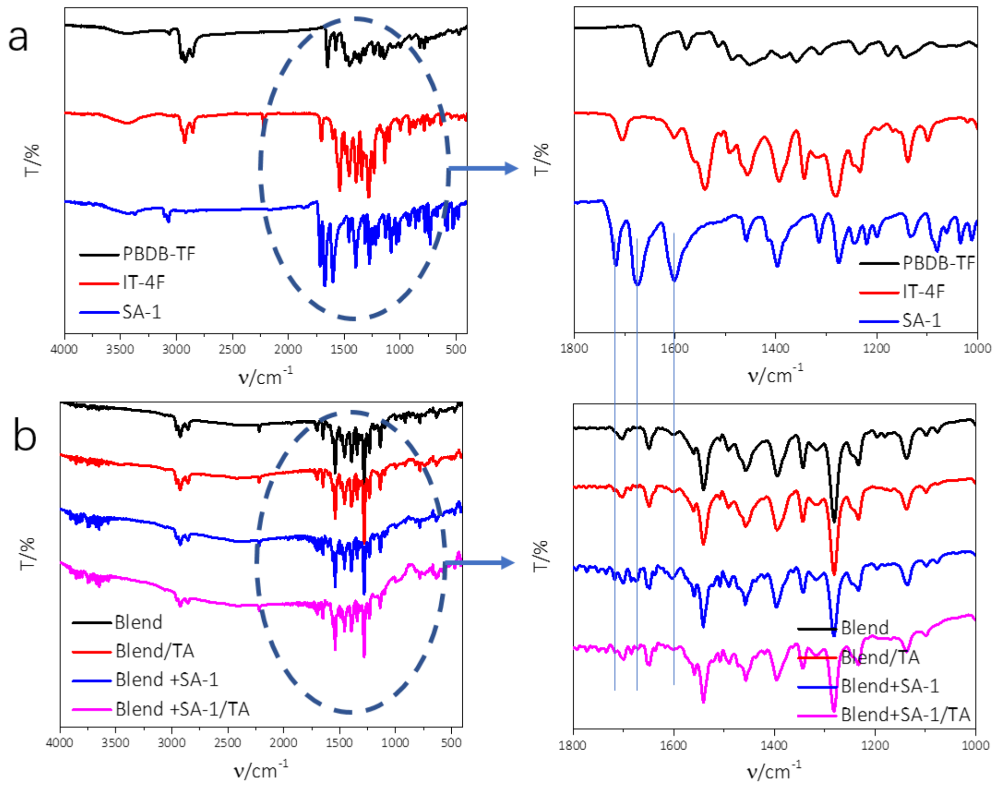

Supplementary Figure 14. **a,** FT-IR spectrum of PBDB-TF, IT-4F and SA-1 recorded in KBr pellets. **b,** IR spectra of PBDB-TF:IT-4F blend processed with different treatments, the samples were fabricated by spin coating the corresponding solution onto the KBr tablets. (First, we investigated the IR spectrum of PBDB-TF, IT-4F and SA-1 solids in the region of 400–4000 cm^{-1} recorded in KBr pellets and magnified the region of 1000 to 1800 cm^{-1} (Supplementary Figure 14a) to make a clear comparison. We found that SA-1 shows intense vibration at about 1716, 1673 and 1601 cm^{-1}. Then we spin-coated the PBDB-TF:IT-4F blend films with different conditions (as-cast, TA, SA-1-added, SA-1-added/TA) onto the KBr tablets and measured the corresponding IR spectrum. As shown in Supplementary Figure 14a and b, the IR spectra of the blend+SA-1 film shows clear peaks at 1716, 1673 and 1601 cm^{-1}. After thermal annealing, these peaks are decreased or disappeared.

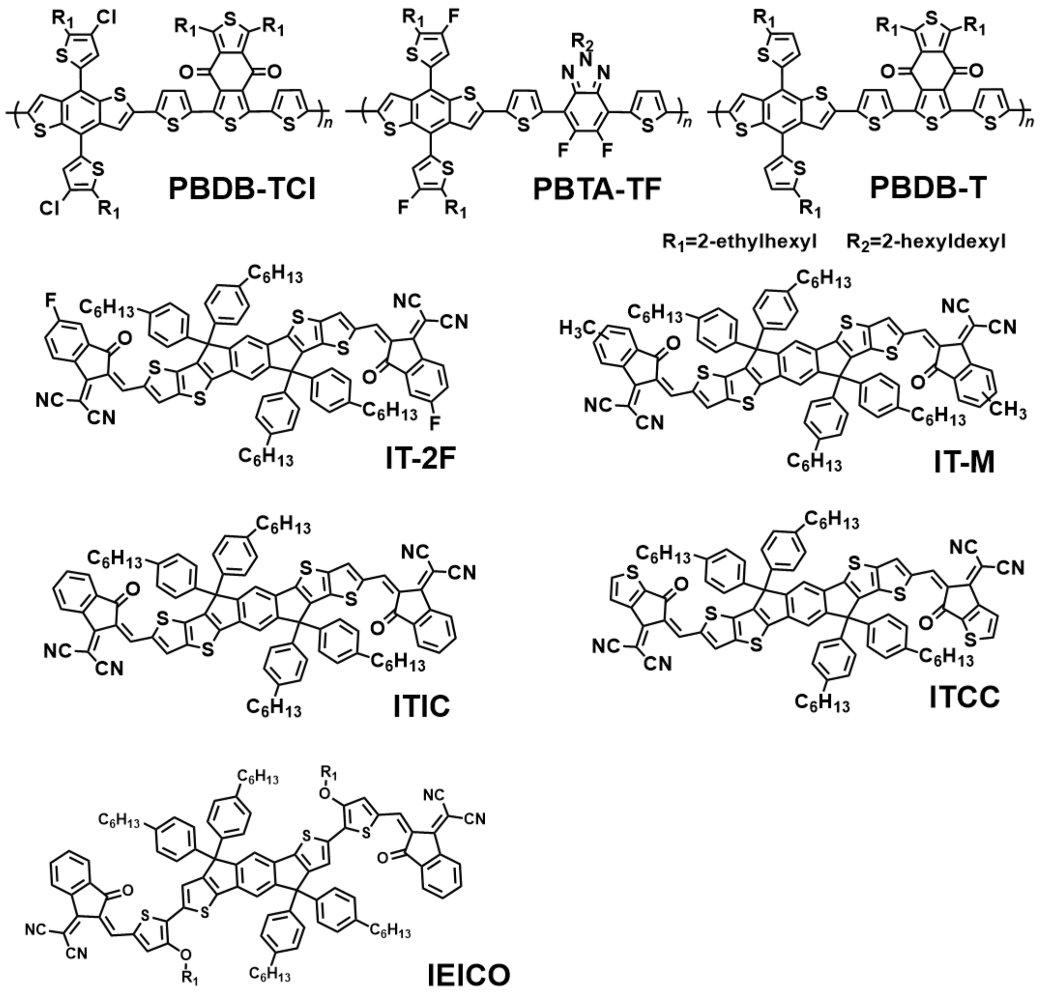

Supplementary Figure 15. Molecular structures of the donor and acceptor materials used for proving SA-1 is a general additive in varied active layers.

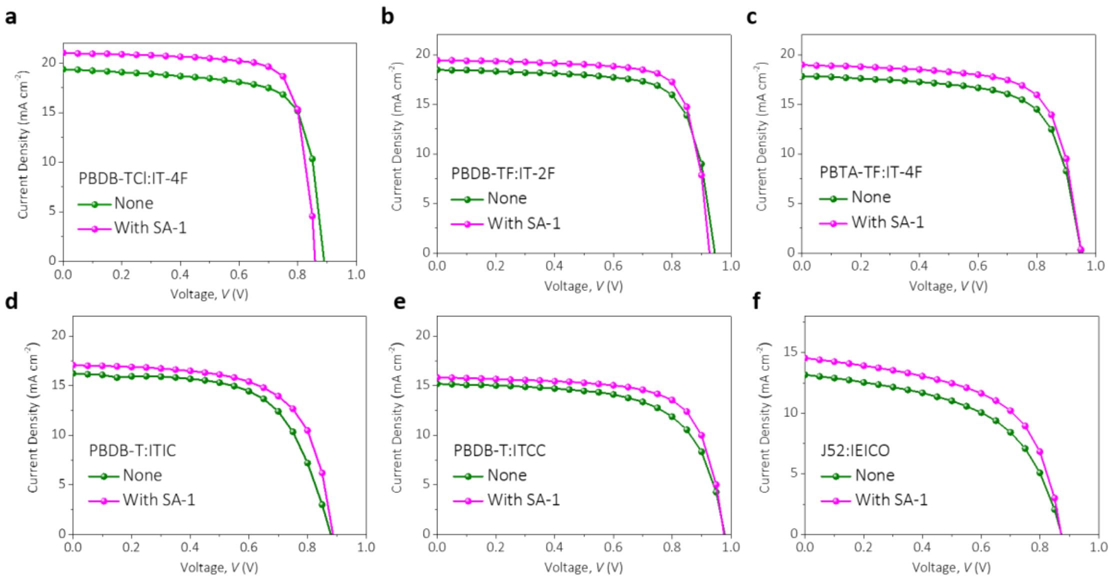

Supplementary Figure 16. *J*–*V* curves of OSC devices processed with and w/o SA-1 based on different active layers, including **a**, PBDB-TF:IT-2F, **b**, PBTA-TF:IT-M, **c**, PBDB-T:ITIC, **d**, PBDB-T:ITCC and **e**, J52:IEICO.

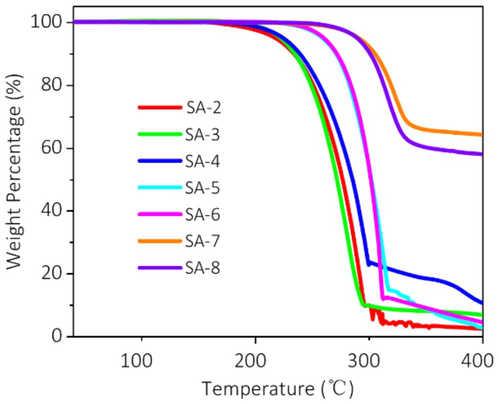

Supplementary Figure 17. TGA plots of SA-x (x=2 to 8) scanned at a rate of 10.0 °C min^{-1}. (The weight of SA-2, SA-3 or SA-4 began to lose about 5% at about 240 °C, similar to SA-1, while SA-5 and SA-6 began at 270 °C, and most of the bulks of SA-x (x=2 to 6) had volatilized to the top substrate of TGA scale. Notably, SA-7 and SA-8 began to loss 5% weight at above 300 °C, and different from other SAs, part of the SA-7 or SA-8 volatilized to the top substrate, while some black solids remained in the scale.)

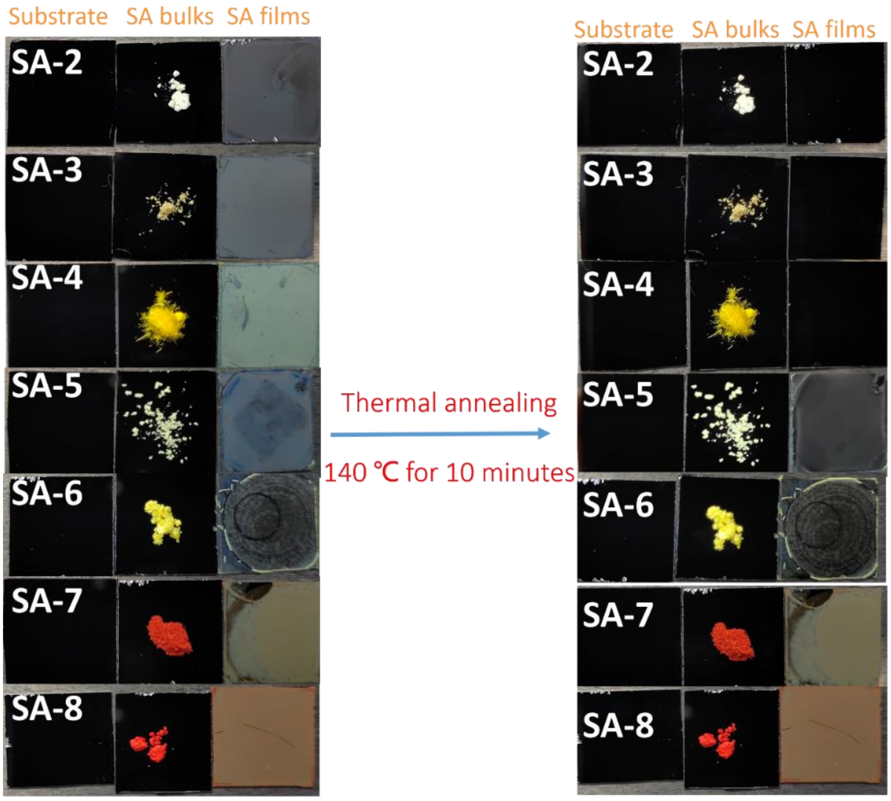

Supplementary Figure 18. Photographs of solid bulks and the respective spin-coated films of seven SAs (SA-2-SA-8) on Fe substrates. Then the films were thermal annealed at 140 °C for 10 minutes. (We found that the SA-x (x=2, 3, and 4) films gradually changed over time and had totally vanished 3 minutes later, and the substrates became clean. For the SA-x (x= 5, 6, 7, and 8), the films changed a little and SA-x remained on the substrates after thermal annealing for 10 minutes. However, for the solid bulks of SA-x, no changes had been seen by naked eye in the whole process, which is consistent with the results obtained from the TGA plot that the bulk of SA-1 exhibited very small weight loss at 140 °C.)

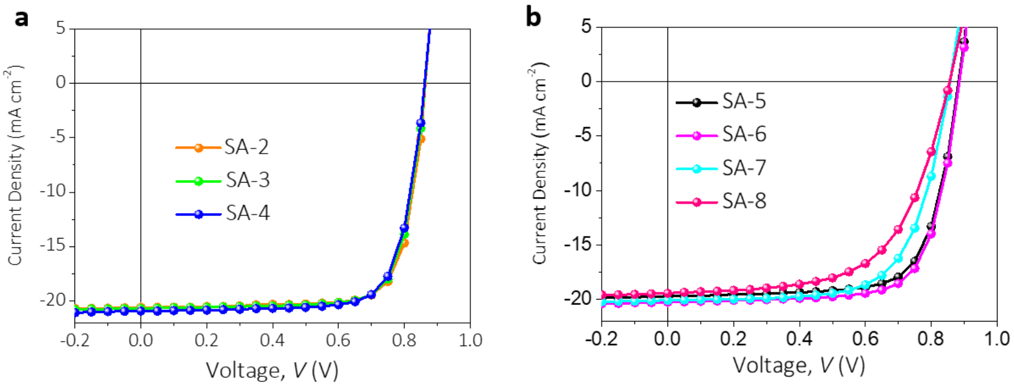

Supplementary Figure 19. *J–V* curves of OSC devices based on PBDB-TF:IT-4F processed with different solid additives.

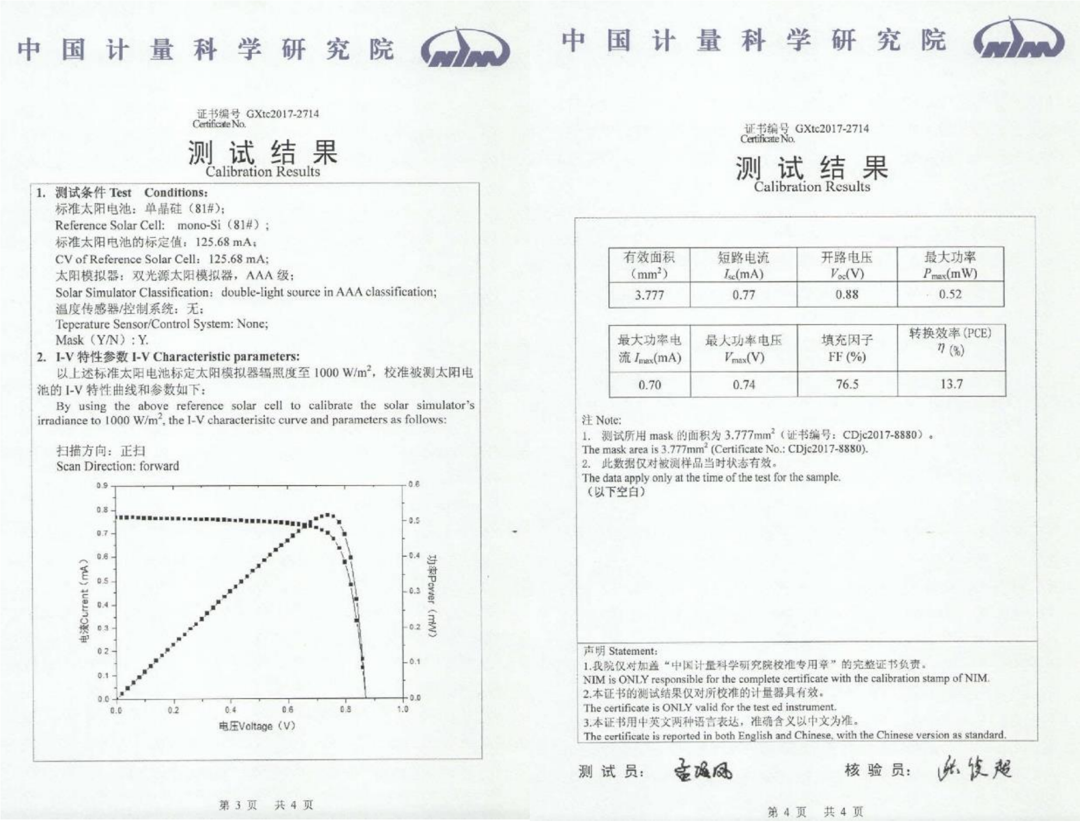

中国计量科学研究院

证书编号 GXtc2017-2714
Certificate No.

测 试 结 果
Calibration Results

1. **测试条件 Test Conditions:**
标准太阳电池：单晶硅（81#）;
Reference Solar Cell: mono-Si（81#）;
标准太阳电池的标定值：125.68 mA;
CV of Reference Solar Cell：125.68 mA;
太阳模拟器：双光源太阳模拟器，AAA 级;
Solar Simulator Classification：double-light source in AAA classification;
温度传感器/控制系统：无;
Teperature Sensor/Control System: None;
Mask（Y/N）: Y.
2. **I-V 特性参数 I-V Characteristic parameters:**
以上述标准太阳电池标定太阳模拟器辐照度至 1000 W/m²，校准被测太阳电池的 I-V 特性曲线和参数如下：
By using the above reference solar cell to calibrate the solar simulator's irradiance to 1000 W/m², the I-V characterisitc curve and parameters as follows:

扫描方向：正扫
Scan Direction: forward

第 3 页 共 4 页

中国计量科学研究院

证书编号 GXtc2017-2714
Certificate No.

测 试 结 果
Calibration Results

有效面积 (mm^2)	短路电流 I_{sc}(mA)	开路电压 V_{oc}(V)	最大功率 P_{max}(mW)
3.777	0.77	0.88	0.52

最大功率电流 I_{max}(mA)	最大功率电压 V_{max}(V)	填充因子 FF (%)	转换效率 (PCE) η (%)
0.70	0.74	76.5	13.7

注 Note:
1. 测试所用 mask 的面积为 3.777mm²（证书编号：CDjc2017-8880）。
The mask area is 3.777mm² (Certificate No.: CDjc2017-8880).
2. 此数据仅对被测样品当时状态有效。
The data apply only at the time of the test for the sample.
（以下空白）

声明 Statement:
1.我院仅对加盖"中国计量科学研究院校准专用章"的完整证书负责。
NIM is ONLY responsible for the complete certificate with the calibration stamp of NIM.
2.本证书的测试结果仅对所校准的计量器具有效。
The certificate is ONLY valid for the test ed instrument.
3.本证书用中英文两种语言表达，准确含义以中文为准。
The certificate is reported in both English and Chinese, with the Chinese version as standard.

测试员： [illegible]　　核验员： [illegible]

第 4 页 共 4 页

Supplementary Figure 20. Test report of the PBDB-TF:IT-4F-based OSCs processed with SA-1 from National Institute of Metrology, China (NIM).

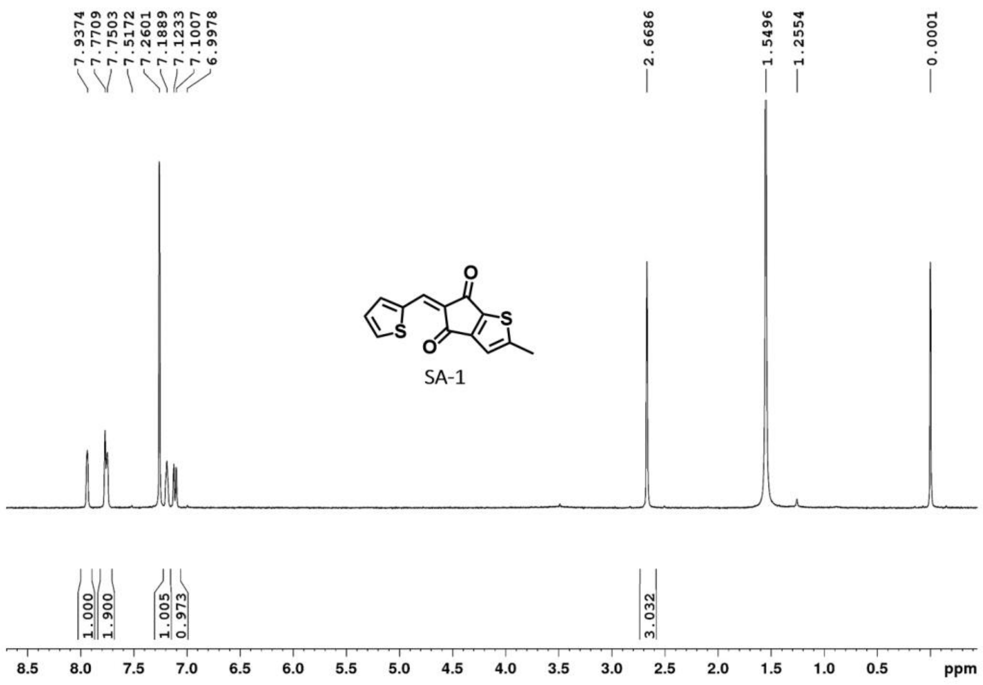

Supplementary Figure 21. ^{1}H NMR spectrum of SA-1.

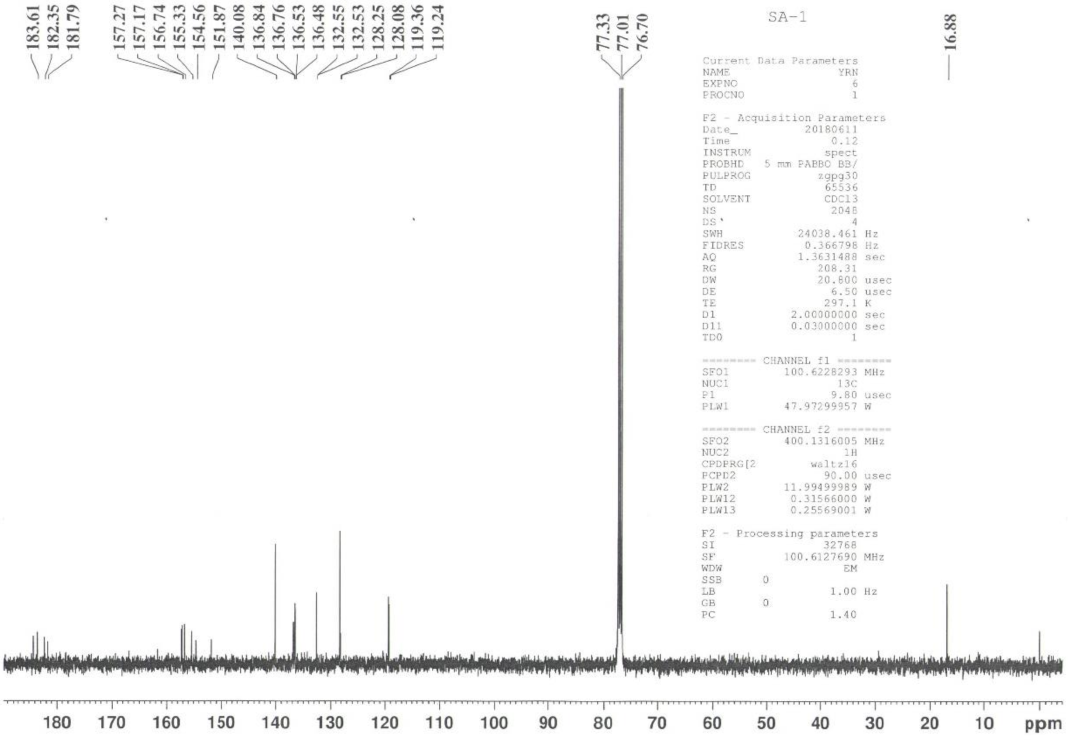

Supplementary Figure 22. ^{13}C NMR spectrum of SA-1.

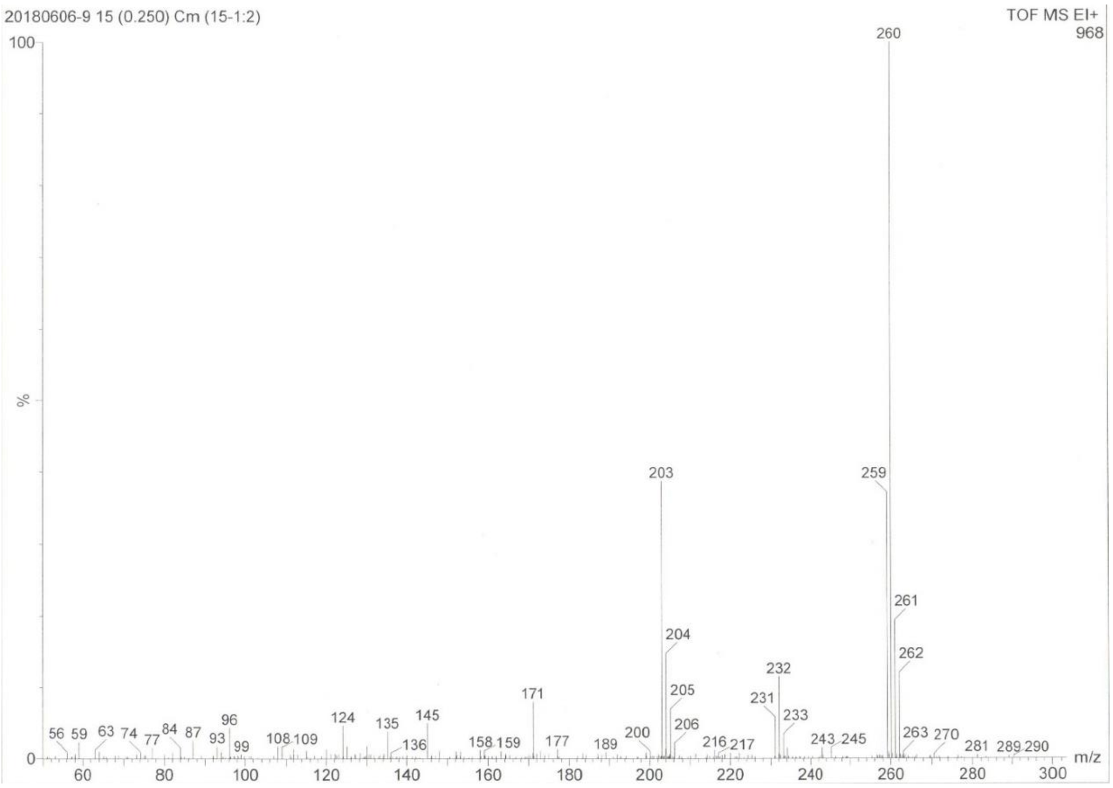

Supplementary Figure 23. MS image of SA-1.

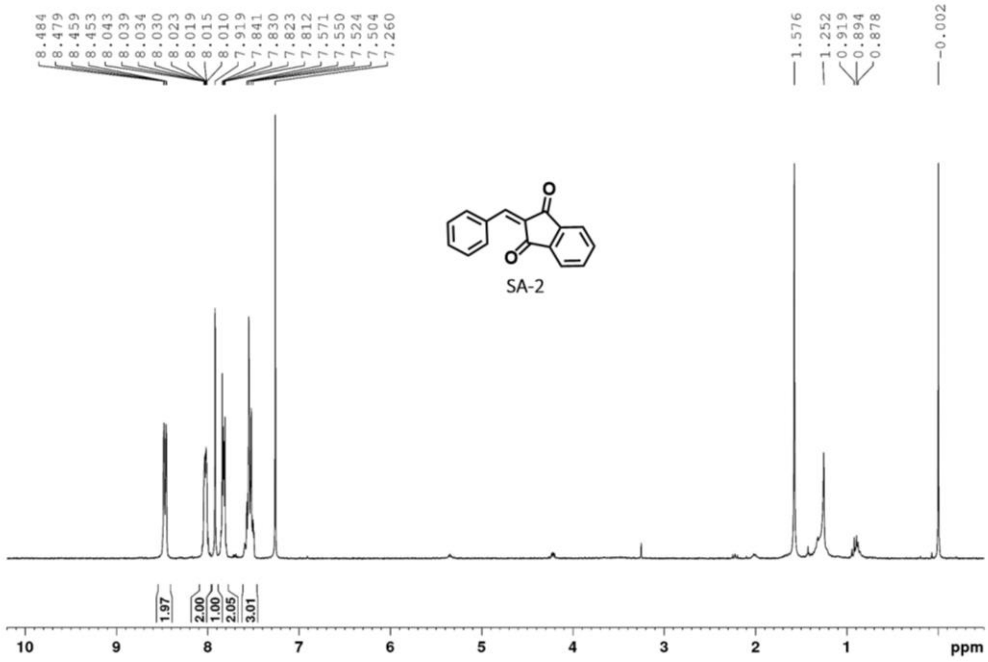

Supplementary Figure 24. ^{1}H NMR spectrum of SA-2.

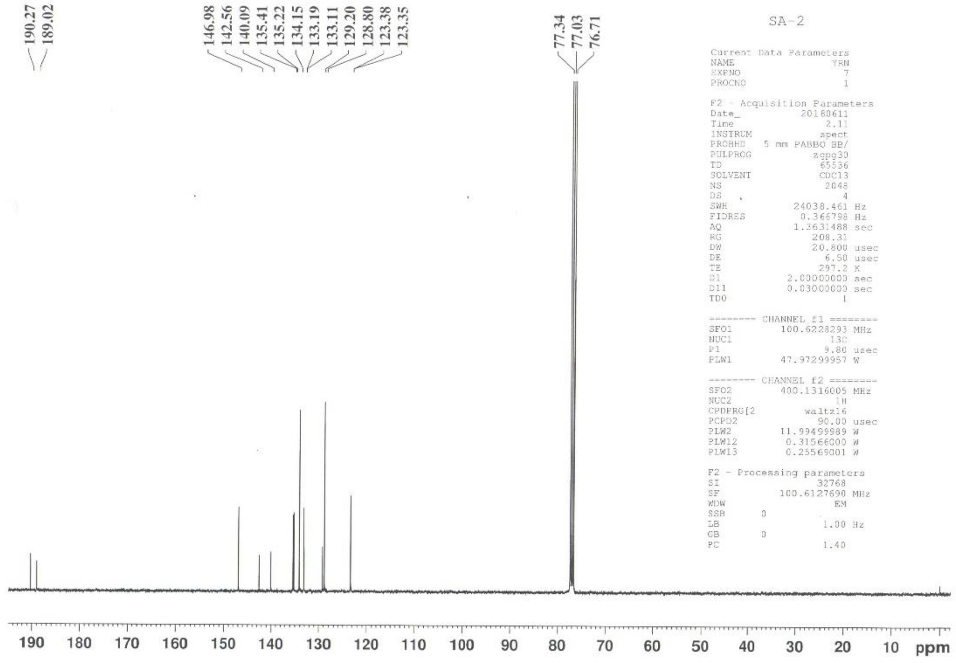

Supplementary Figure 25. ^{13}C NMR spectrum of SA-2.

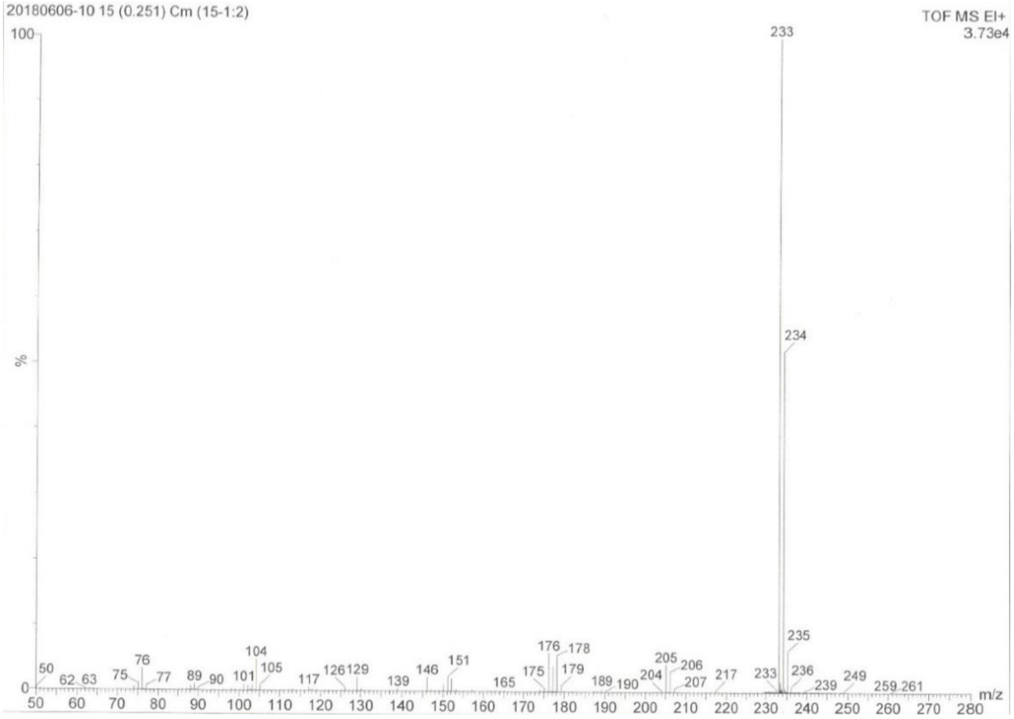

Supplementary Figure 26. MS image of SA-2.

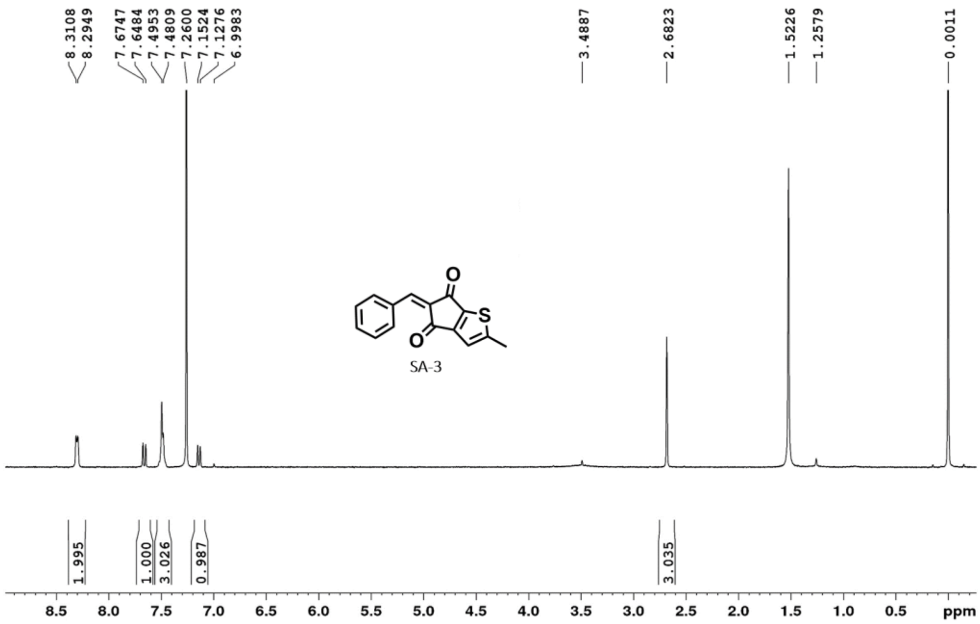

Supplementary Figure 27. ^{1}H NMR spectrum of SA-3.

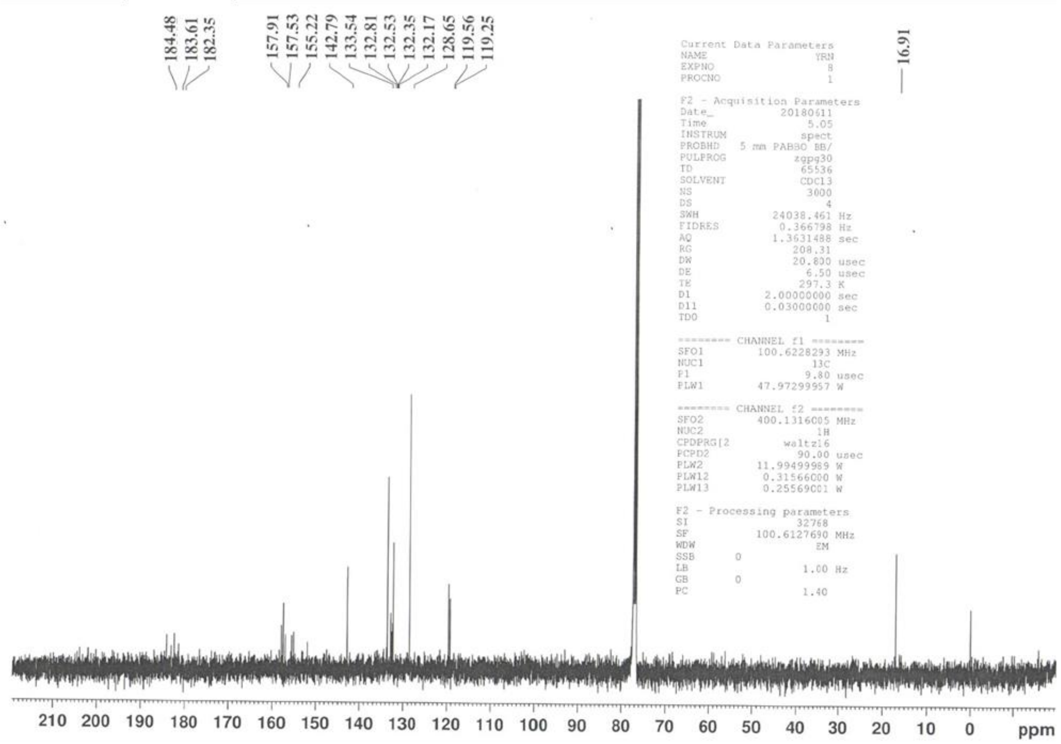

Supplementary Figure 28. ^{13}C NMR spectrum of SA-3.

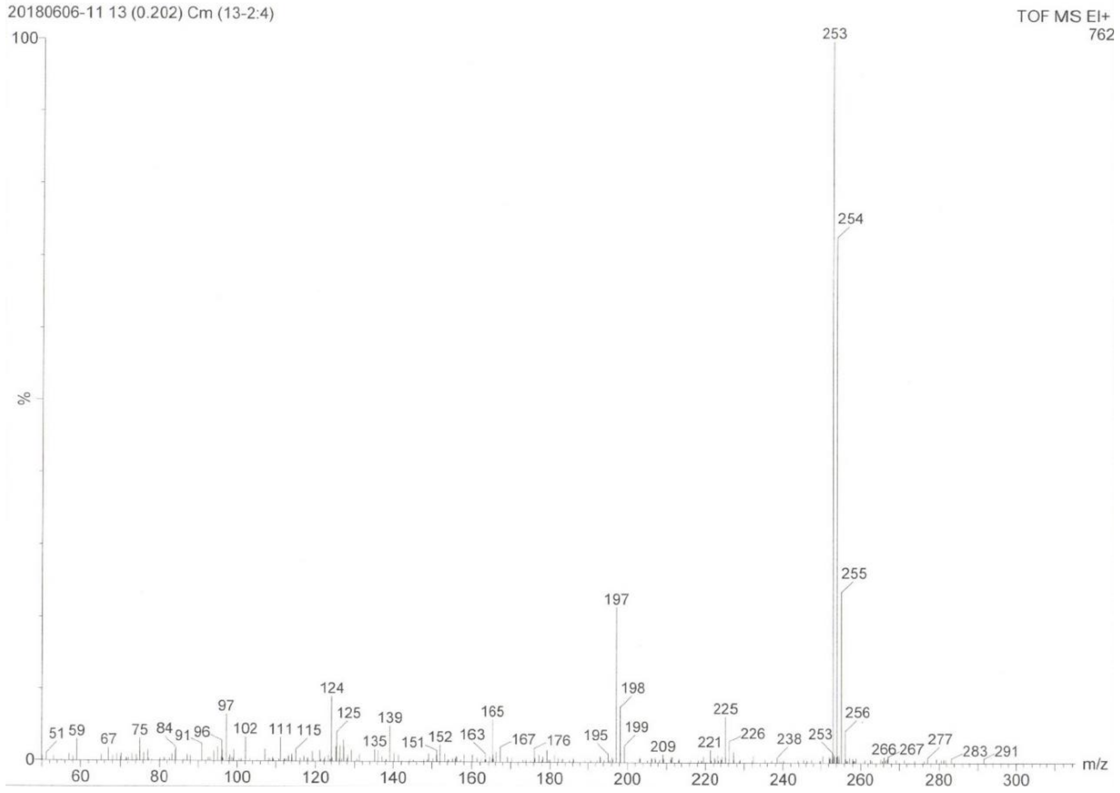

Supplementary Figure 29. MS image of SA-3.

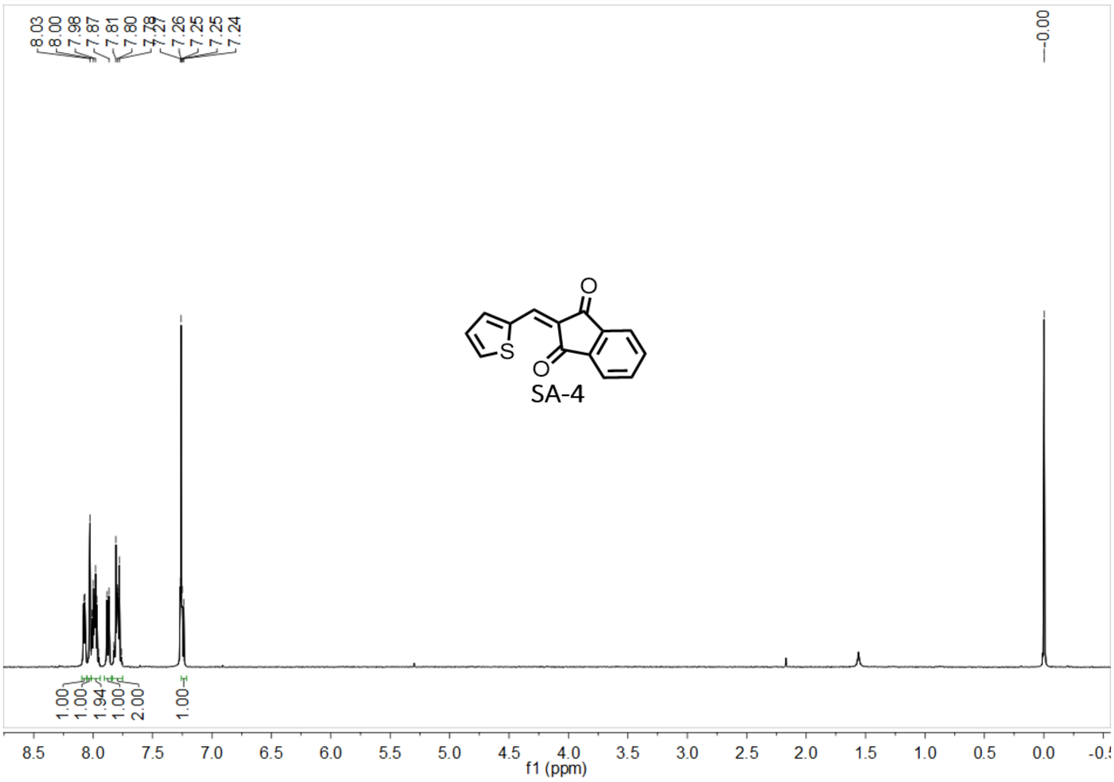

Supplementary Figure 30. ^{1}H NMR spectrum of SA-4.

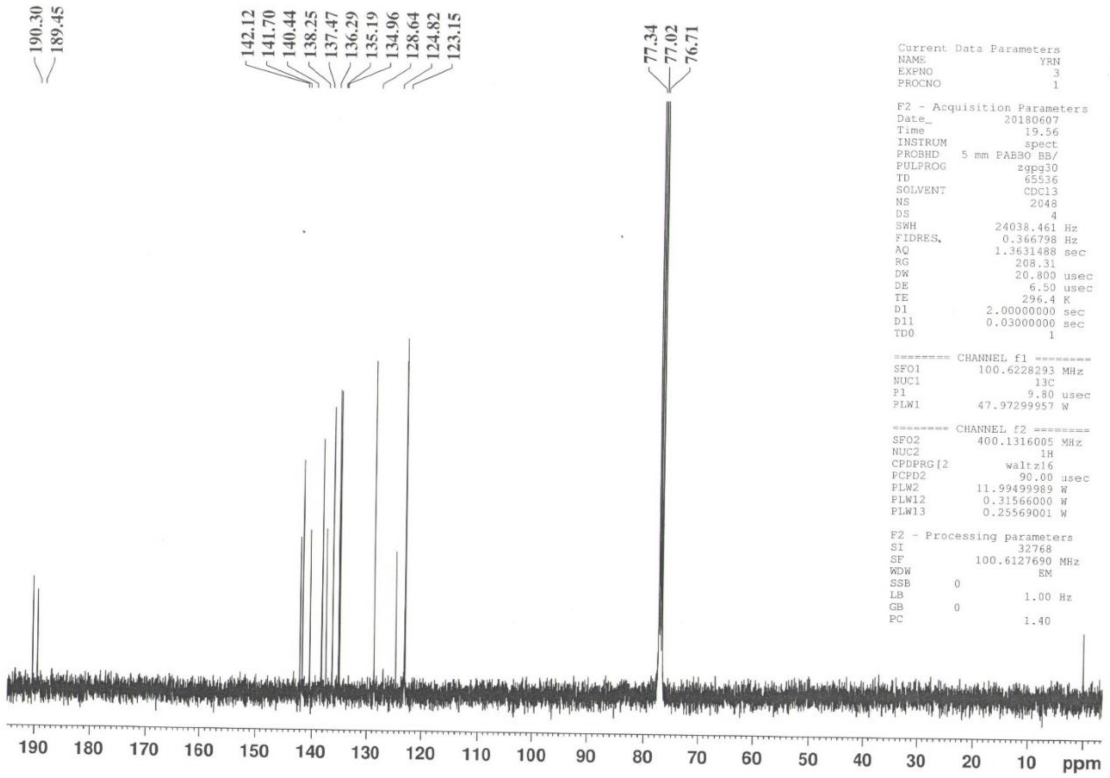

Supplementary Figure 31. ^{13}C NMR spectrum of SA-4.

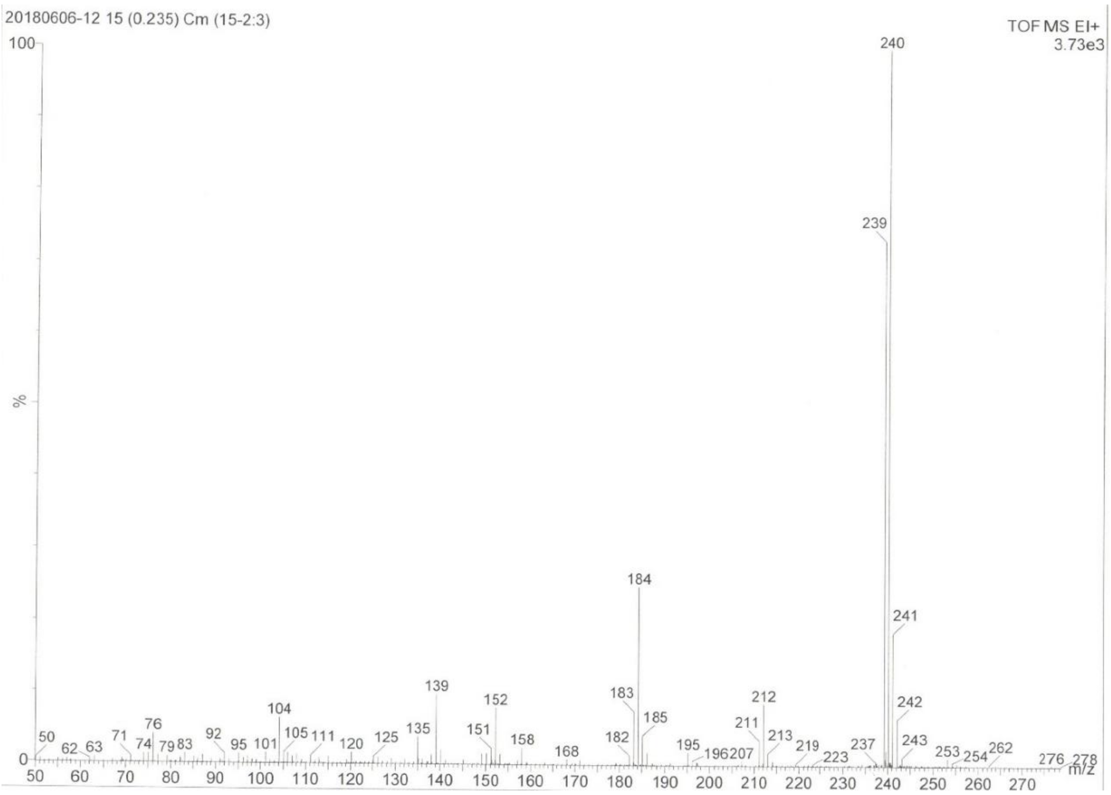

Supplementary Figure 32. MS image of SA-4.

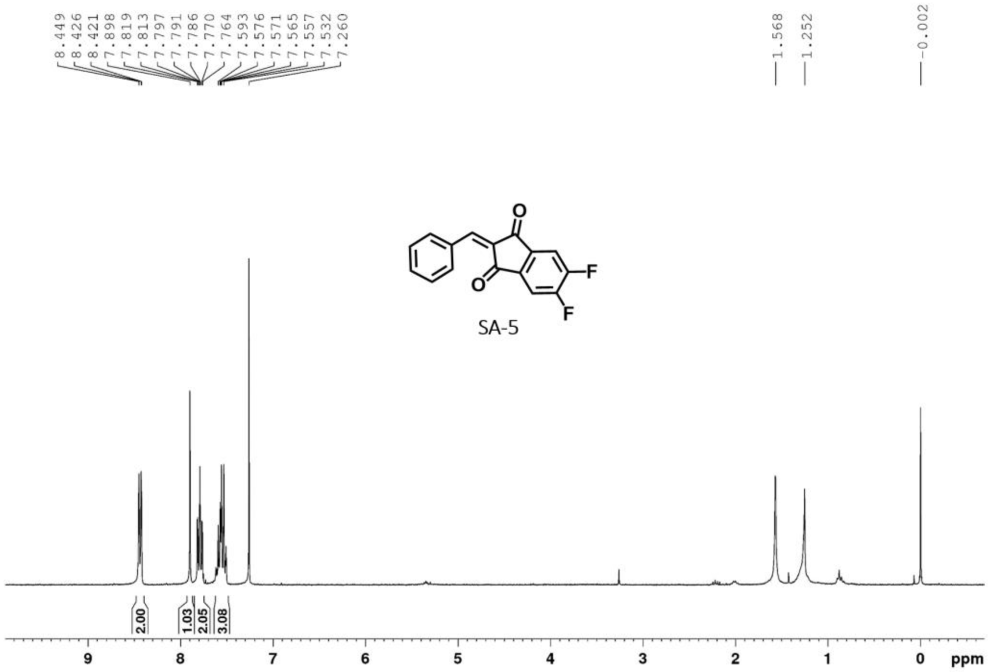

Supplementary Figure 33. ^{1}H NMR spectrum of SA-5.

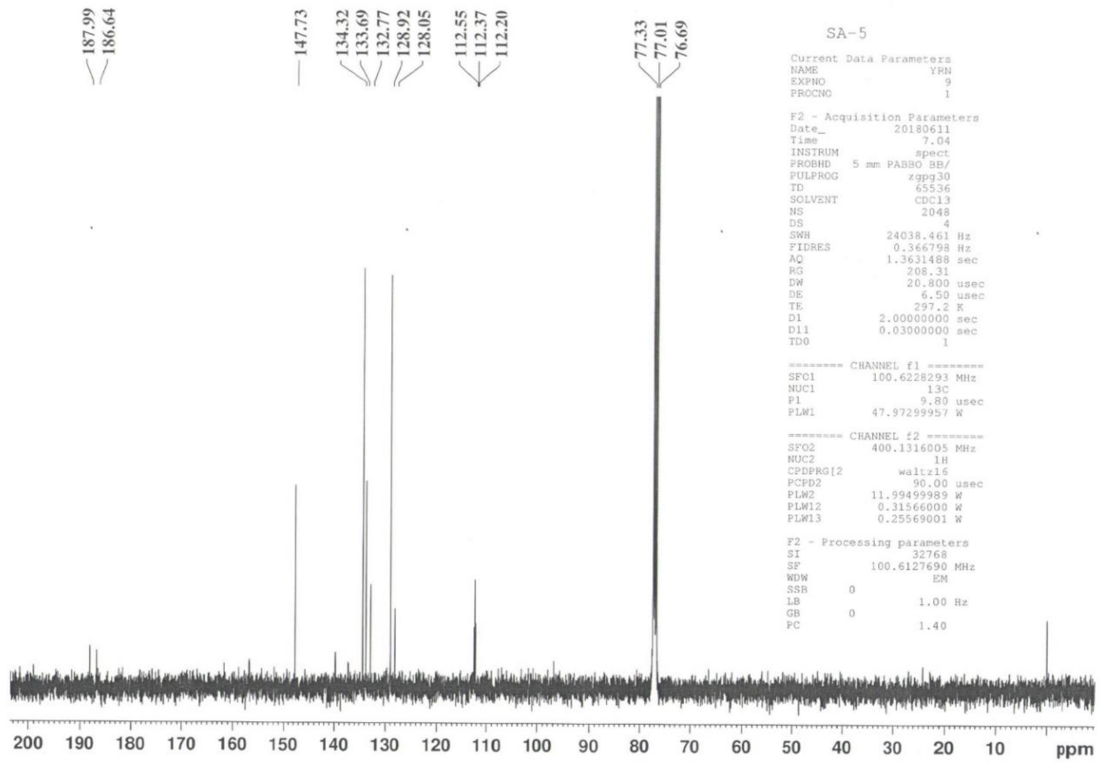

Supplementary Figure 34. ^{13}C NMR spectrum of SA-5.

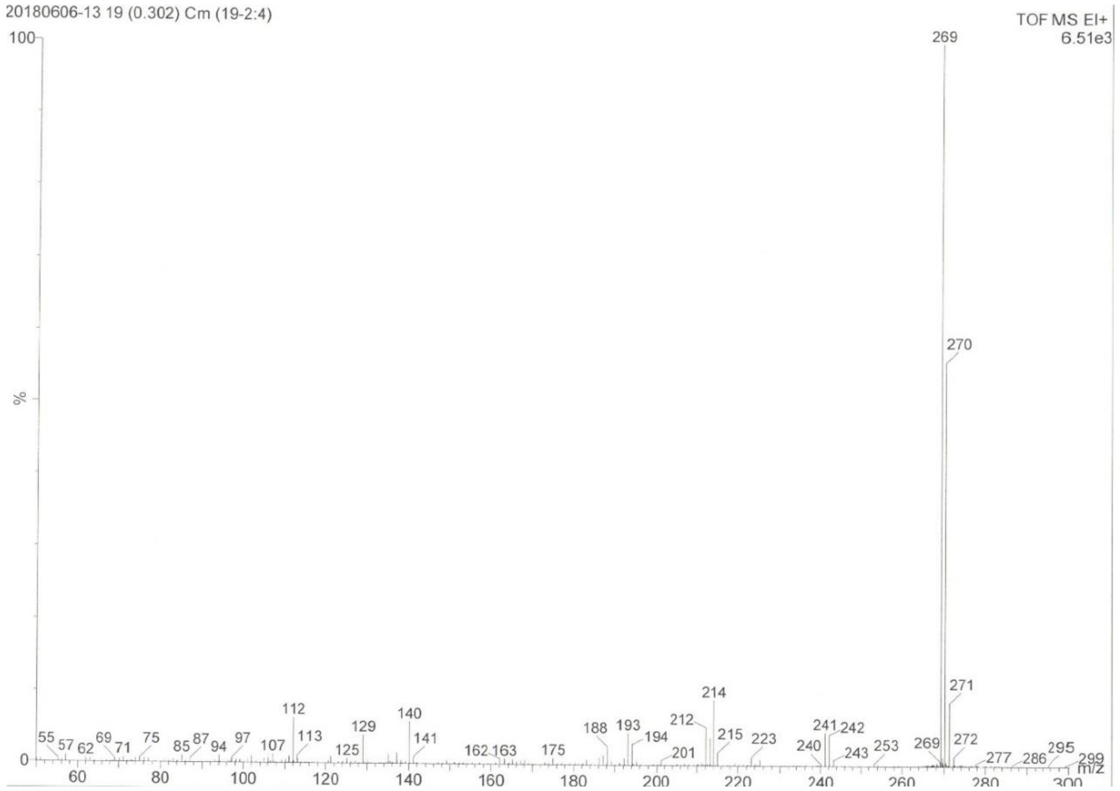

Supplementary Figure 35. MS image of SA-5.

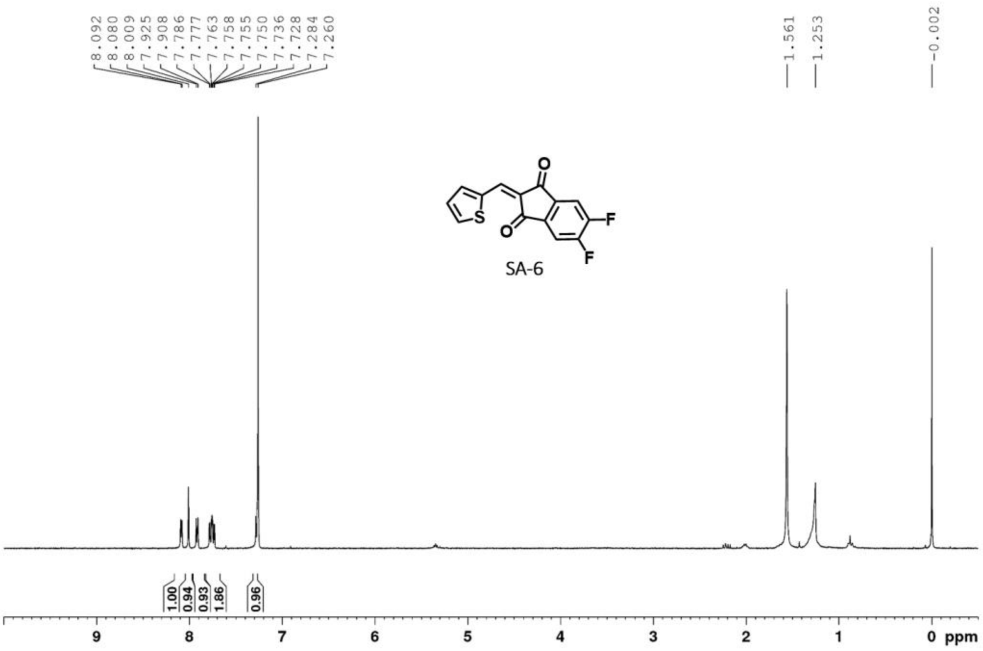

Supplementary Figure 36. ^{1}H NMR spectrum of SA-6.

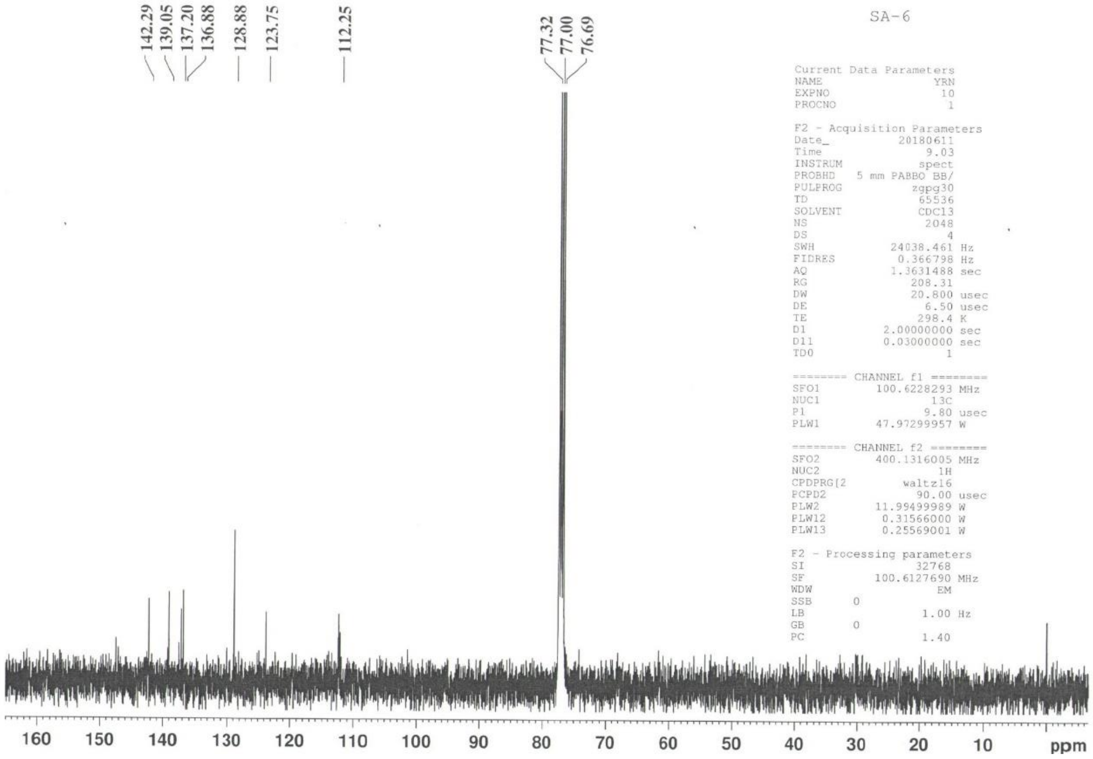

Supplementary Figure 37. ^{13}C NMR spectrum of SA-6.

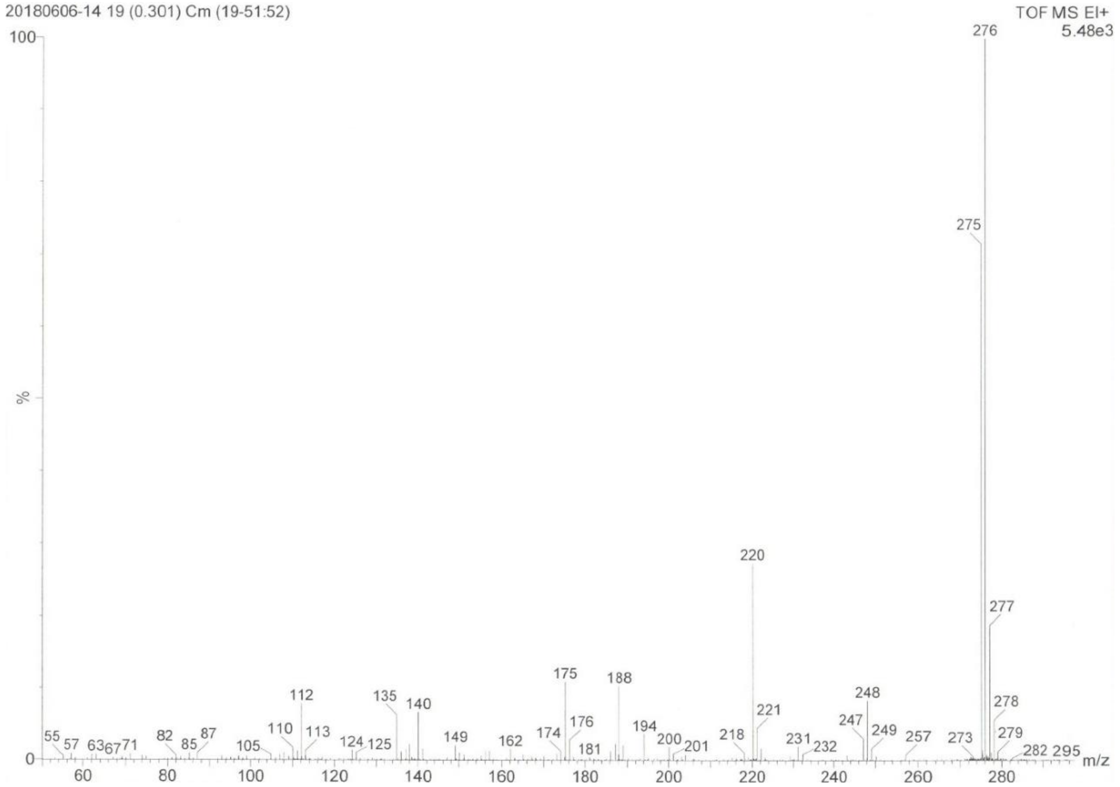

Supplementary Figure 38. MS image of SA-6.

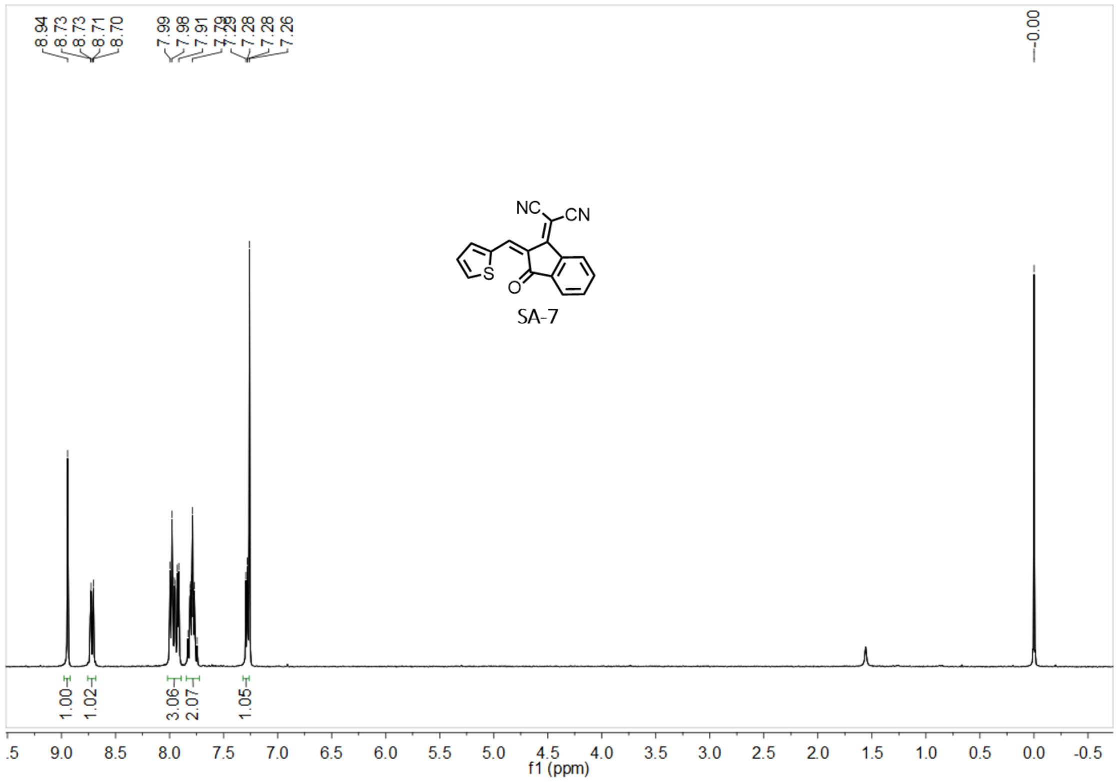

Supplementary Figure 39. ^{1}H NMR spectrum of SA-7.

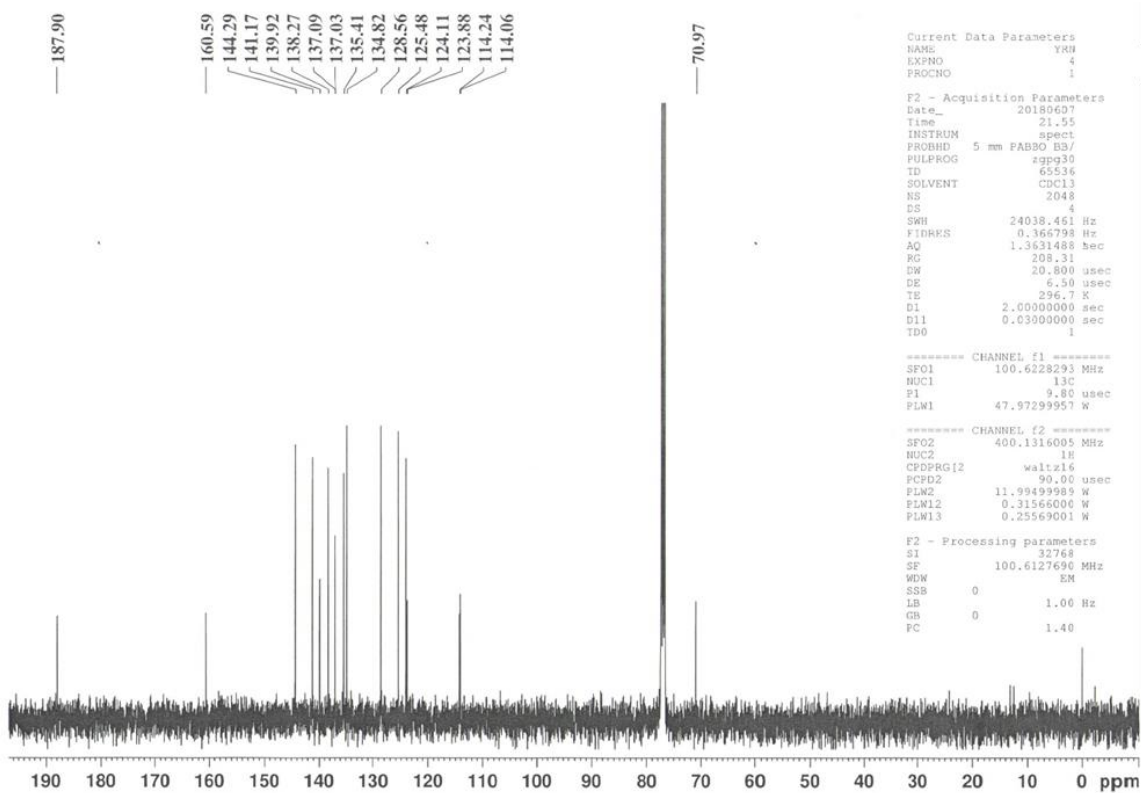

Supplementary Figure 40. ^{13}C NMR spectrum of SA-7.

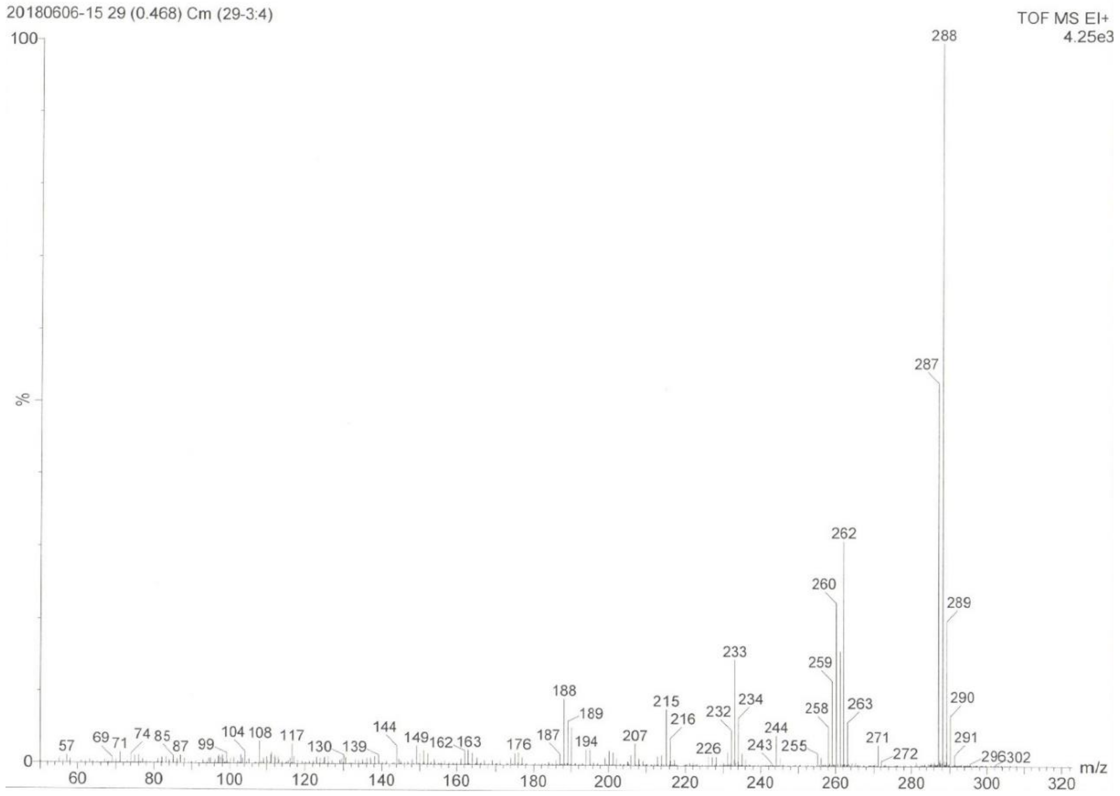

Supplementary Figure 41. MS image of SA-7.

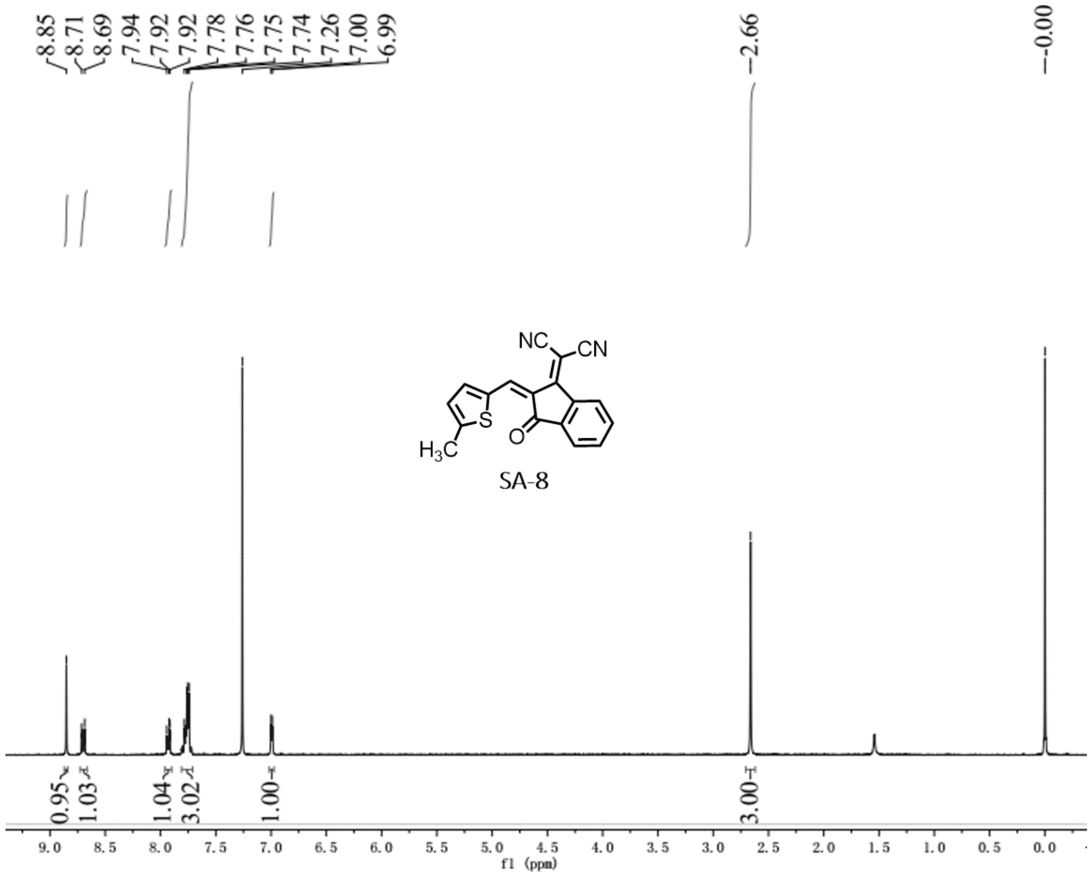

Supplementary Figure 42. ^{1}H NMR spectrum of SA-8.

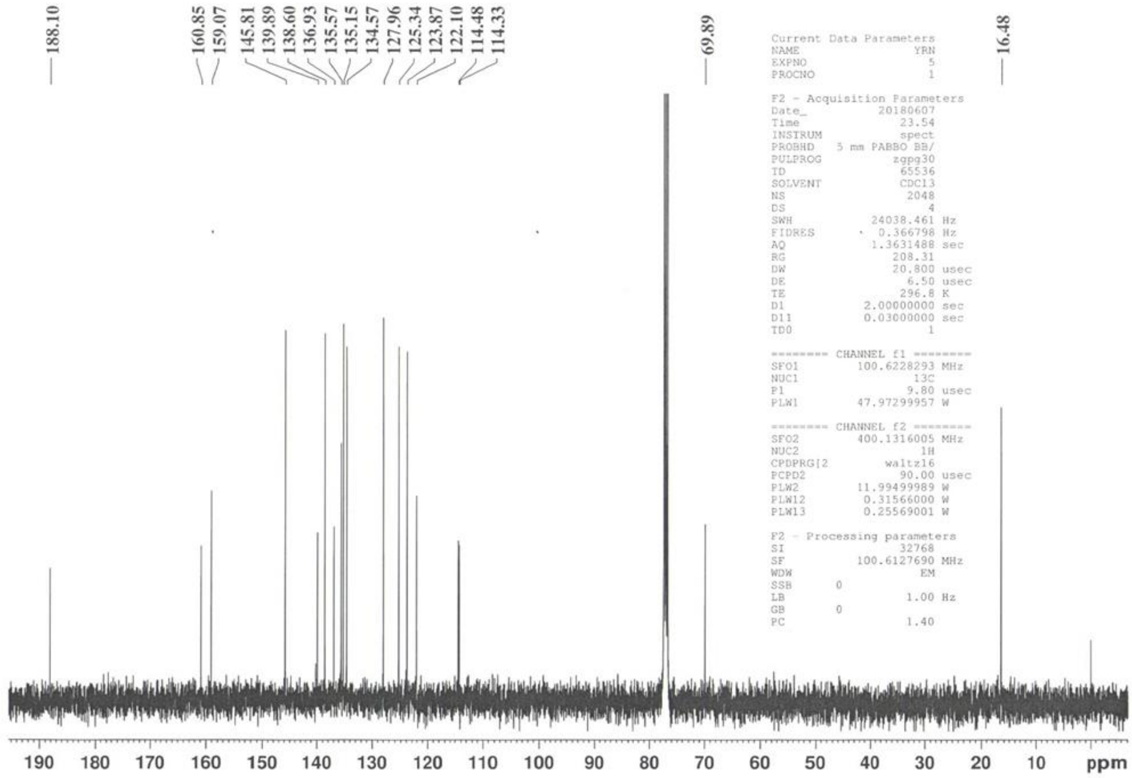

Supplementary Figure 43. ^{13}C NMR spectrum of SA-8.

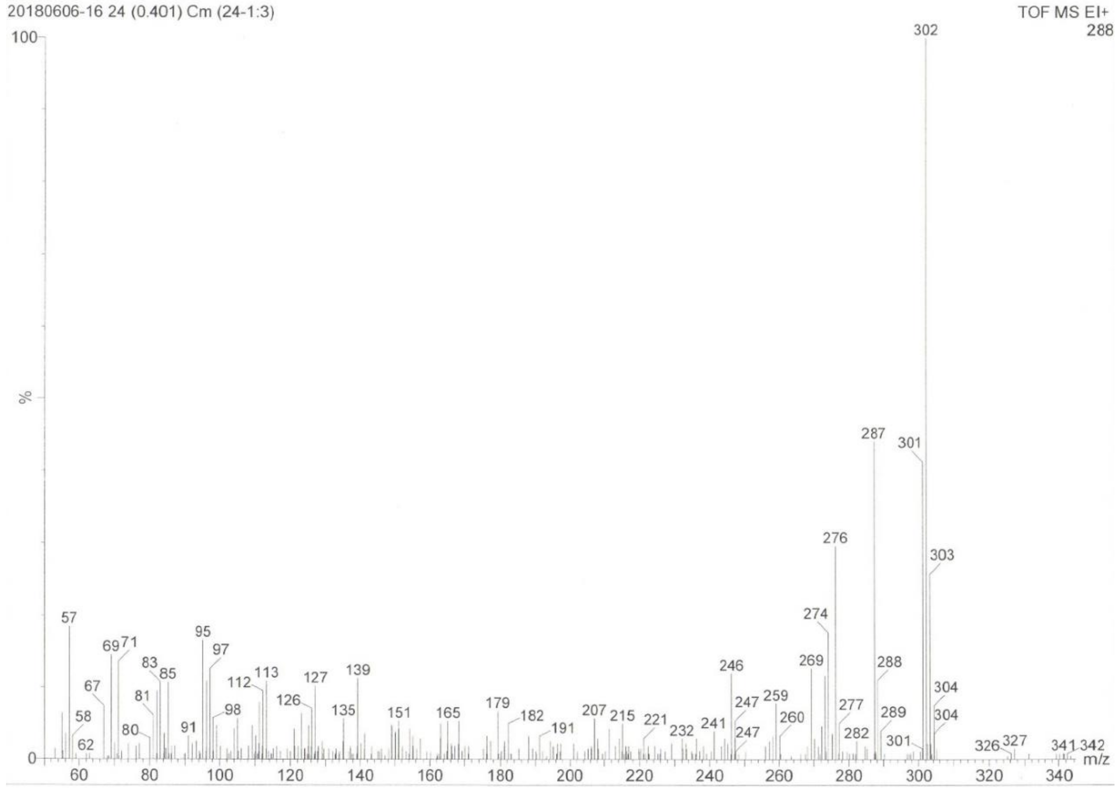

Supplementary Figure 44. MS image of SA-8.

Supplementary Tables

Supplementary Table 1. Photovoltaic parameters for PBDB-TF:IT-4F based devices with varying mole ratios of IT-4F:SA-1 incorporated into the casting solution (the weight ratio of PBDB-TF:IT-4F is kept as 1:1 and the devices are processed with thermal annealing).

IT-4F:SA-1 mol/mol	Mol.wt. wt.%[a]	V_{oc} (V)	J_{sc} (mA cm^{-2})	FF	PCE (%)
1:0	0	0.89 ± 0.01	18.6 ± 0.5 (18.0)	0.71 ± 0.02	11.8± 0.3
1:0.05	0.86	0.89 ± 0.01	18.7 ± 0.6(18.2)	0.72 ± 0.01	12.0± 0.2
1:0.1	1.73	0.89 ± 0.01	19.0 ± 0.4 (18.8)	0.72 ± 0.01	12.2± 0.2
1:0.5	8.6	0.88 ± 0.01	19.9 ± 0.3 (19.4)	0.75 ± 0.03	13.0± 0.3
1:1	17.3	0.87 ± 0.01	20.3± 0.2 (20.1)	0.76 ± 0.01	13.4± 0.2
1:1.5	25.9	0.86 ± 0.01	20.0 ± 0.3 (19.5)	0.75 ± 0.02	13.2 ± 0.2
1:2	34.6	0.85 ± 0.01	18.3± 0.4 (17.7)	0.73 ± 0.02	11.3 ± 0.4
1:3	51.9	0.83 ± 0.01	14.7± 0.5 (14.2)	0.65 ± 0.02	8.0 ± 0.5

[a)] Integrated current density calculated from EQE curves are shown in the parentheses.

Supplementary Table 2. The photovoltaic parameters of the PSCs based on PBDB-TF:IT-4F processed with or w/o SA-1 at different film thicknesses.

Additive	V_{oc} (V)	J_{sc} (mA cm^{-2})	FF	PCE (%)	Thickness (nm)
None	0.89 ± 0.01	18.9 ± 0.3	0.71 ± 0.02	11.8 ± 0.3	100 ± 10
	0.88 ± 0.01	19.1 ± 0.2	0.69 ± 0.01	11.5 ± 0.2	150 ± 10
	0.88 ± 0.01	18.9 ± 0.3	0.67 ± 0.02	11.1 ± 0.4	200 ± 10
	0.88 ± 0.01	21.2 ± 0.2	0.59 ± 0.03	11.1 ± 0.3	250 ± 10

	0.87 ± 0.01	20.4 ± 0.5	0.55 ± 0.01	9.8 ± 0.3	300 ± 10
	0.87 ± 0.01	20.0 ± 0.2	0.53 ± 0.01	9.2 ± 0.2	350 ± 10
	0.86 ± 0.01	17.5 ± 0.3	0.44 ± 0.02	6.9 ± 0.4	400 ± 10
	0.87 ± 0.01	20.3 ± 0.2	0.76 ± 0.01	13.3 ± 0.3	100 ± 10
	0.86 ± 0.01	20.4 ± 0.6	0.75 ± 0.02	13.2 ± 0.2	150 ± 10
	0.85 ± 0.01	21.1 ± 0.2	0.70 ± 0.02	12.6 ± 0.3	200 ± 10
With SA-1	0.85 ± 0.01	22.1± 0.1	0.67 ± 0.02	12.5 ± 0.2	250 ± 10
	0.84 ± 0.01	22.4 ± 0.5	0.67 ± 0.03	12.6 ± 0.4	300 ± 10
	0.84 ± 0.01	21.9 ± 0.3	0.67 ± 0.02	12.3 ± 0.2	350 ± 10
	0.83 ± 0.01	22.1 ± 0.3	0.66 ± 0.03	12.1 ± 0.4	400 ± 10

Supplementary Table 3. The best photovoltaic parameters for PBDB-TF:IT-4F based devices processed with four different conditions including as-cast, TA, SA-1-added, SA-1-added/TA.

Fabrication conditions	V_{oc} (V)	J_{sc} (mA cm^{-2})	FF	PCE (%)
As-cast	0.90	18.7	0.69	11.6
TA	0.89	19.1	0.72	12.2
SA-1-added	0.90	20.2	0.61	11.1
SA-1-added/TA	0.87	20.4	0.78	13.8

Supplementary Table 4. Photovoltaic parameters for PBDB-TF:IT-4F based devices processed with SA-1 or SA-7 under different thermal annealing temperatures.

Additive	TA temperature (°C)	V_{oc} (V)	J_{sc} (mA cm^{-2})	FF	PCE (%)

17.3% SA-1	100	0.90	19.4	0.66	11.5
	120	0.88	19.6	0.70	12.1
	140	0.87	20.4	0.77	13.7
	160	0.86	20.0	0.75	12.9
	180	0.75	19.7	0.74	10.9
	200	0.72	18.5	0.73	9.8
19.2% SA-7	100	0.85	19.2	0.58	9.5
	120	0.85	19.6	0.59	9.8
	140	0.84	19.8	0.70	11.6
	160	0.84	20.1	0.73	12.1
	180	0.76	19.9	0.74	11.2
	200	0.71	19.0	0.72	9.6

Supplementary Table 5. Electron and hole mobility for SCLC measurements and the photo-CELIV mobility.

Fabrication condition	μ_e [cm^2 V^{-1} s^{-1}]	μ_h [cm^2 V^{-1} s^{-1}]	photo-CELIV mobility [cm^2 V^{-1} s^{-1}]
IT-4F/TA	2.0×10^{-4}	-	-
IT-4F+SA-1/TA	1.4×10^{-3}	-	-
PBDB-TF:IT-4F	1.3×10^{-4}	-	4.9×10^{-5}
PBDB-TF:IT-4F/TA	1.7×10^{-4}	3.2×10^{-4}	7.7×10^{-5}
PBDB-TF:IT-4F+SA-1	1.4×10^{-4}	-	6.8×10^{-5}
PBDB-TF:IT-4F+SA-1/TA	1.3×10^{-3}	3.5×10^{-4}	1.2×10^{-4}

Supplementary Table 6. Detailed optical properties of PBDB-TF, IT-4F and their blend films under different treatments, including as-cast films, TA films, SA-1-added

films and SA-1-added TA films.

Fabrication conditions	PBDB-TF		IT-4F		PBDB-TF[1]:IT-4F[2] blend	
	λ_{max} (nm)	ε_{max} (cm^{-1})	λ_{max} (nm)	ε_{max} (cm^{-1})	$\lambda_{max1}/\lambda_{max2}$ (nm)	$\varepsilon_{max1}/\varepsilon_{max2}$ (cm^{-1})
As-cast	617	1.05×10^5	718	1.11×10^5	626/718	$8.36\times10^4/6.36\times10^4$
TA	620	1.08×10^5	718	1.12×10^5	627/718	$8.45\times10^4/6.63\times10^4$
SA-1	620	1.06×10^5	716	1.12×10^5	627/714	$8.36\times10^4/6.58\times10^4$
SA-1+TA	620	1.09×10^5	730	1.20×10^5	627/728	$8.49\times10^4/6.86\times10^4$

Supplementary Table 7. Photovoltaic parameters for PBDB-TF:IT-4F based devices processed with DIO or SA-1 under different preparation conditions.

Additive	Preparation condition[a)]	V_{oc} (V)	J_{sc} (mA cm^{-2})	FF	PCE (%)
0.5% DIO	Device 1	0.86 ± 0.01	20.1 ± 0.2	0.75 ± 0.02	13.3 ± 0.2
	Device 2	0.84 ± 0.02	18.1 ± 2.2	0.67 ± 0.06	9.3 ± 1.3
17.3% SA-1	Device 1	0.87 ± 0.01	20.2 ± 0.3	0.76 ± 0.01	13.4 ± 0.2
	Device 2	0.86 ± 0.01	20.4 ± 0.3	0.75 ± 0.01	13.2 ± 0.3

[a)] For the Device 1, thermal annealing of active layer film was directly carried out after the film casting, while Device 2 was annealed after 24 hours' standing.

Supplementary Notes

Supplementary Note 1: *Synthesis of SA-1*

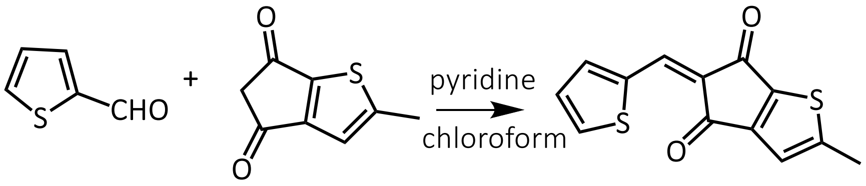

1 SA-1

Compound 1 was synthesized as reported. [5] Thenaldehyde (0.11 g, 1 mmol) and compound 1 (0.73 g, 5 mmol) were added to a mixture solvent of chloroform (30 mL) and pyridine (1 mL). Then the reaction was placed in an oil bath at 65 °C and stirred for 48 hours. The mixture was firstly purified by silica gel column chromatography by using dichloromethane as eluent and then recrystallized by ethanol to offer the product as white solid (199 mg, yield 85%). .**^{1}H NMR** (400 MHz, CDCl3, δ): 7.94 (s, 1H), 7.76 (d, *J* = 8.2 Hz, 2H), 7.19 (s, 1H), 7.11 (d, *J* = 9.0 Hz, 1H), 2.67 (s, 3H). **^{13}C NMR** (100 MHz, $CDCl_3$): δ (ppm): 183.61, 182.35, 181.79, 157.27, 157.17, 156.74, 155.33, 154.56, 151.87, 140.08, 136.84, 136.76, 136.53, 136.48, 132.55, 132.53, 128.25, 128.08, 119.36, 119.24, 16.88. MS-EI: m/z = 260 (M^+) Analytical calculation for $C_{13}H_8O_2S_2$: C 59.98, H 3.10. Experimental result: C 59.94, H 3.20 (See Supplementary Figure 21 to 23).

Supplementary Note 2: *Synthesis of SA-2*

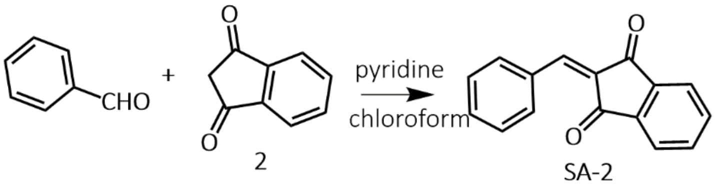

2 SA-2

Benzaldehyde (0.1 g, 1 mmol) and Compound 2(0.86 g, 5 mmol) were added to a mixture solvent of chloroform (30 mL) and pyridine (1 mL). Then the reaction was placed in an oil bath at 65 °C and stirred for 48 hours. The mixture was firstly purified

by silica gel column chromatography by using dichloromethane as eluent and then recrystallized by petroleum ether to offer the product as yellow solid (187 mg, yield 82%). .^{1}**H NMR** (300 MHz, CDC13, δ): 8.47 (dd, *J* = 7.7, 1.5 Hz, 2H), 8.08 – 7.98 (m, 2H), 7.92 (s, 1H), 7.86 – 7.79 (m, 2H), 7.60 – 7.49 (m, 3H). ^{13}**C NMR** (100 MHz, $CDCl_3$): δ (ppm): 190.27, 189.02, 146.98, 142.56, 140.09, 135.41, 135.22, 134.15, 133.19, 133.11, 129.20, 128.80, 123.38, 123.35. MS-EI: m/z = 233 (M^+) Analytical calculation for $C_{16}H_{10}O_2$: C 82.04, H 4.30. Experimental result: C 81.84, H 4.36 (See Supplementary Figure 24 to 26).

Supplementary Note 3: *Synthesis of SA-3*

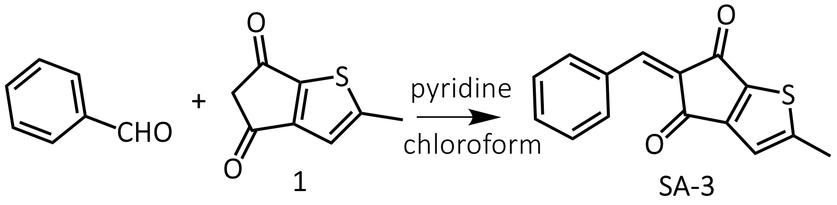

Benzaldehyde (0.1 g, 1 mmol) and compound 1 (0.86 g, 5 mmol) were added to a mixture solvent of chloroform (30 mL) and pyridine (1 mL). Then the reaction was placed in an oil bath at 65 °C and stirred for 48 hours. The mixture was firstly purified by silica gel column chromatography by using dichloromethane as eluent and then recrystallized by ethanol to offer the product as yellow solid (120 mg, yield 51%).^{1}**H NMR** (400 MHz, CDC13, δ): 8.30 (d, J = 8 Hz 2H), 7.65(d, J = 10 Hz 1H), 7.48 (m, 3H), 7.14 (d, J = 10 Hz, 1H), 2.68 (s, 3H). ^{13}**C NMR** (100 MHz, $CDCl_3$): δ (ppm): 184.48, 183.61, 182.35, 157.91, 157.53, 155.22, 142.79, 133.54, 132.81, 132.53, 132.35, 132.17, 128.65, 119.56, 119.25, 16.91. MS-EI: m/z = 253 (M+) Analytical calculation for C15H10O2S: C 70.85, H 3.96. Experimental result: C 70.88, H 3.41

(See Supplementary Figure 27 to 29).

Supplementary Note 4: *Synthesis of SA-4*

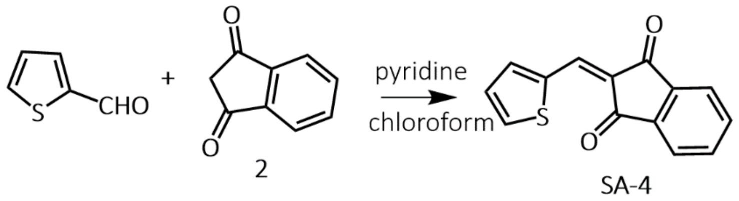

Thenaldehyde (0.11 g, 1 mmol) and Compound 2(0.86 g, 5 mmol) were added to a mixture solvent of chloroform (30 mL) and pyridine (1 mL). Then the reaction was placed in an oil bath at 65 °C and stirred for 48 hours. The mixture was firstly purified by silica gel column chromatography by using dichloromethane as eluent and then recrystallized by ethanol to offer the product as yellow solid (189 mg, yield 79%). .^{1}**H NMR** (300 MHz, CDCl3, δ): 8.07 (d, J = 3.1 Hz, 1H), 8.03 (s, 1H), 8.02 – 7.94 (m, 2H), 7.88 (d, J = 5.0 Hz, 1H), 7.84 – 7.75 (m, 2H), 7.26 – 7.21 (m, 1H). ^{13}**C NMR** (100 MHz, $CDCl_3$): δ (ppm): 190.30, 189.45, 142.12, 141.70, 140.44, 138.25, 137.47, 136.29, 135.19, 134.96, 135.19, 134.96, 128.64, 124.82, 123.15. MS-EI: m/z = 240 (M^+) Analytical calculation for $C_{14}H_8O_2S$: C 69.98, H 3.36. Experimental result: C 69.73, H 3.43 (See Supplementary Figure 30 to 32).

Supplementary Note 5: *Synthesis of SA-5*

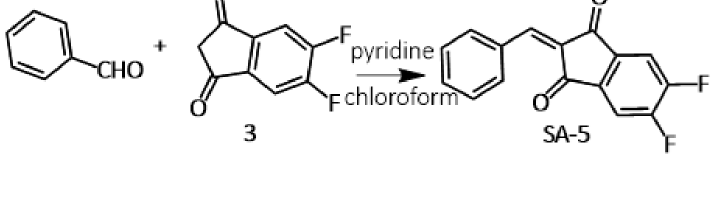

Benzaldehyde (0.1 g, 1 mmol) and Compound 3(0.99 g, 5 mmol) were added to a mixture solvent of chloroform (30 mL) and pyridine (1 mL). Then the reaction was placed in an oil bath at 65 °C and stirred for 48 hours. The mixture was firstly purified

by silica gel column chromatography by using dichloromethane as eluent and then recrystallized by ethanol to offer the product as faint yellow solid (194 mg, yield 72%). .[1]**H NMR** (300 MHz, CDCl3, δ): 8.51 – 8.40 (m, 2H), 7.90 (s, 1H), 7.84 – 7.74 (m, 2H), 7.63 – 7.49 (m, 3H). [13]**C NMR** (100 MHz, $CDCl_3$): δ (ppm): 187.99, 186.64, 143.73, 134.32, 133.69, 132.77, 128.92, 128.05, 112.55, 112.37, 112.20. MS-EI: m/z = 269 (M^+) Analytical calculation for $C_{16}H_8F_2O_2$: C 71.11, H 2.98. Experimental result: C 70.82, H 3.05. (See Supplementary Figure 33 to 45)

Supplementary Note 6: *Synthesis of SA-6*

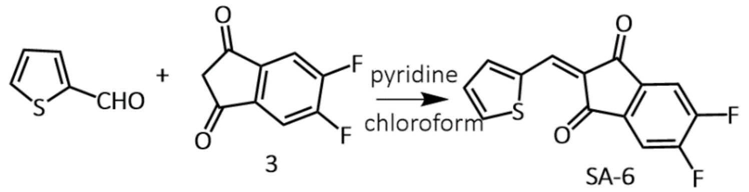

Thenaldehyde (0.11 g, 1 mmol) and Compound 3(0.99 g, 5 mmol) were added to a mixture solvent of chloroform (30 mL) and pyridine (1 mL). Then the reaction was placed in an oil bath at 65 °C and stirred for 48 hours. The mixture was firstly purified by silica gel column chromatography by using dichloromethane as eluent and then recrystallized by ethanol to offer the product as yellow solid (193 mg, yield 70%). . [1]**H NMR** (300 MHz, $CDCl_3$, δ): 8.09 (d, *J* = 3.8 Hz, 1H), 8.01 (s, 1H), 7.92 (d, *J* = 5.0 Hz, 1H), 7.76 (ddd, *J* = 8.2, 6.8, 2.6 Hz, 2H), 7.28 (s, 1H). [13]**C NMR** (100 MHz, $CDCl_3$): δ (ppm): 142.29, 139.05, 137.20, 136.88, 128.88, 123.75. MS-EI: m/z = 276 (M^+) Analytical calculation for $C_{16}H_8F_2O_2$: C 71.11, H 2.98. Experimental result: C 70.84, H 3.05 (See Supplementary Figure 36 to 38).

Supplementary Note 7: *Synthesis of SA-7*

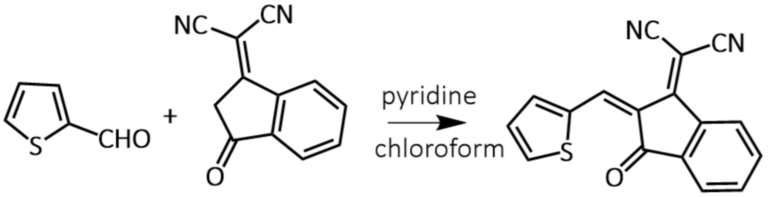

4

SA-7

Thenaldehyde (0.11 g, 1 mmol) and Compound 4(0.97 g, 5 mmol) were added to a mixture solvent of chloroform (30 mL) and pyridine (1 mL). Then the reaction was placed in an oil bath at 65 °C and stirred for 48 hours. The mixture was firstly purified by silica gel column chromatography by using dichloromethane as eluent and then recrystallized by ethanol to offer the product as orange solid (193 mg, yield 70%). **^{1}H NMR** (300 MHz, $CDCl_3$, δ): 8.94 (s, 1H), 8.72 (dd, *J* = 6.4, 1.9 Hz, 1H), 8.02 – 7.89 (m, 3H), 7.84 – 7.72 (m, 2H), 7.32 – 7.26 (m, 1H). **^{13}C NMR** (100 MHz, $CDCl_3$): δ (ppm):187.90, 160.59, 144.29, 141.17, 139.92, 138.27, 137.09, 137.03, 135.41, 134.82, 128.56, 125.48, 124.11, 123.88, 114.24, 114.06, 70.97. MS-EI: m/z = 288 (M^+) Analytical calculation for $C_{17}H_8N_2OS$: C 70.82, H 2.80. Experimental result: C 70.35, H 2.83, N 9.69 (Supplementary Figure 39 to 41).

Supplementary Note 8: *Synthesis of SA-8*

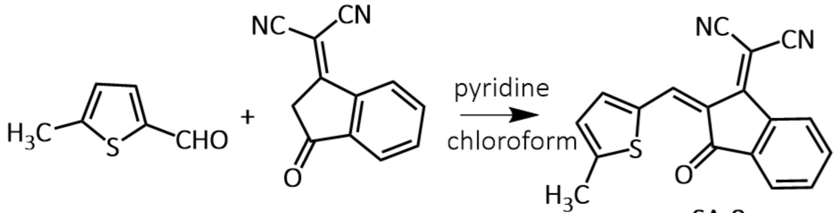

5 4 SA-8

Compound 5 (0.13 g, 1 mmol) and Compound 4(0.97 g, 5 mmol) were added to a mixture solvent of chloroform (30 mL) and pyridine (1 mL). Then the reaction was placed in an oil bath at 65 °C and stirred for 48 hours. The mixture was firstly purified

by silica gel column chromatography by using dichloromethane as eluent and then recrystallized by ethanol to offer the product as orange solid (214 mg, yield 71%). **^{1}H NMR** (300 MHz, $CDCl_3$, δ): 8.85 (s, 1H), 8.70 (dd, J = 6.3, 2.0 Hz, 1H), 7.93 (dd, J = 5.9, 2.5 Hz, 1H), 7.81 – 7.71 (m, 3H), 6.99 (d, J = 3.3 Hz, 1H), 2.66 (s, 3H). **^{13}C NMR** (100 MHz, CDCl3): δ (ppm): 188.10, 160.85, 159.07, 145.81, 139.89, 138.60, 136.93, 135.57, 135.15, 134.57, 127.96, 125.34, 123.87, 122.10, 114.48, 114.33, 69.89, 16.48. MS-EI: m/z = 302 (M^+) Analytical calculation for $C_{18}H_{10}N_2OS$: C 71.51, H 3.33, N 9.27. Experimental result: C 71.13, H 3.19, N 9.35 (See Supplementary Figure 42 to 44).

Supplementary Note 9: *Materials*

Polymer PBDB-TF[3] and small molecule IT-4F[4] were prepared according to the reported literatures. Herein, PBDB-TF exhibits a M_n of 14.3 kDa with a polydispersity index (PDI) of 2.3. PFN-Br, Compound 2, Compound 3, Compound 4, and Compound 5 were purchased from Solarmer Materials (Beijing) Inc. All the other reagents and solvents were purchased from Sigma Aldrich, Alfa Aesar Co., TCI chemical Co., Thermo Fisher Scientific Inc. and used without further purification.

Supplementary Methods

Optical absorption and electro chemical properties. Absorption spectra of diluted solutions (dissolved in chloroform) and films (spin-coated on quartz substrates) were measured on a Hitachi UH4150 UV-Vis spectrophotometer. The films with or without SA-1 were spin-coated from the respective solutions at the same spin speed and had almost the same thickness. The molecular energy levels measurements were carried out by measuring the CV of SA-1 in CH_2Cl_2 solution (1mM) containing Tetrabutylammonium hexa-fluorophosphate (Bu4NPF6, 0.1 M) as the supporting electrolytes on a CHI650D electrochemical workstation with three electrodes system (glassy-carbon, platinum-wire and Ag/Ag^+ electrode as working electrode, counter electrode, and reference electrode) as electrolyte. The voltages were referenced externally to ferrocene (Fc) redox couple (4.80 eV below vacuum) and the scan rate was 50 mV/s.

Material characterization. The ^{1}H NMR spectra were recorded on a BRUKER Fourier 300 spectrometer in CDCl3 at 293 K. Thermogravimetric analysis (TGA) was measured on a Pyris 1 TGA under a nitrogen flow. Differential scanning calorimetry (DSC) measurements were performed using a TA Instruments differential scanning calorimeter (Q2000) under nitrogen at a heating rate of 10 °C min^{-1}.

SCLC charge mobility measurement. The hole-only and electron-only device structures of ITO/PEDOT:PSS/pristine or blend films /Au and ITO/ZnO/ pristine or blend films /Al were used to perform the mobility measurements, respectively. The mobilities of the blend film were measured by the SCLC method, and the equation J =

$(9/8)\varepsilon_o\varepsilon_r\mu_e V^2/L^3$ was used to calculate the mobilities, where ε_o is the vacuum permittivity, ε_r is the dielectric constant of the polymer, μ is the charge carrier mobility, V is the effective applied voltage, and L is the thickness of the film.

Photo-CELIV. The charge carrier mobility (μ) is calculated using the following equation (1):

$$\mu = \frac{2d^2}{3At_{\max}{}^2[1+0.36\frac{\Delta j}{j(0)}]} \; if\, \Delta j \le j(0)$$

$$\mu = \frac{2d^2}{3At_{\max}{}^2} \; if\, \Delta j > j(0) \qquad (1)$$

where d is the active layer thickness, A is the voltage rise speed, t_{max} is the time corresponding to the maximum of the extraction peak, and *j(0)* is the displacement current.

Lifetime testing. The devices were tested continuously for 130 hours on a Photovoltaic Performance Decay Testing System (Model: PVLT-6001M-16A) under the maximum output conditions.

Supplementary References

1. Yao, H., *et al.* A Wide Bandgap Polymer with Strong π-π Interaction for Efficient Fullerene-Free Polymer Solar Cells. *Adv. Energy Mater.* **6**, 1600742 (2016).
2. Frisch, M. J., *et al.* Gaussian 09. Gaussian, Inc. (2009).
3. Zhang, M., Guo, X., Ma, W., Ade, H., Hou, J. A Large-Bandgap Conjugated Polymer for Versatile Photovoltaic Applications with High Performance. *Adv. Mater.* **27**, 4655-4660 (2015).
4. Zhao, W., *et al.* Molecular Optimization Enables over 13% Efficiency in Organic Solar Cells. *J. Am. Chem. Soc.* **139**, 7148-7151 (2017).
5. Cui, Y., *et al.* Fine-Tuned Photoactive and Interconnection Layers for Achieving over 13% Efficiency in a Fullerene-Free Tandem Organic Solar Cell. *J. Am. Chem. Soc.* **139**, 7302-7309 (2017).
